# Supplementary material for: Monetary incentives and peer referral in promoting secondary distribution of HIV self-testing among men who have sex with men in China: A randomized controlled trial
Source: PLoS Med. 2022 Feb 14;19(2):e1003928. doi: 10.1371/journal.pmed.1003928 (PMC8887971; doi:10.1371/journal.pmed.1003928)
Supplement: S1 Table — (DOCX) [file pmed.1003928.s001.docx]

**S1 Table. Characteristics of the index participants regarding 3-month follow-up survey completion, 2019-2020 (N=309)**

|  | **Index participants completed the 3-month follow-up survey** | |
| --- | --- | --- |
|  | **Yes**  **(n=293)** | **No**  **(n=16)** |
| **Age (years)** |  |  |
| *≤ 30* | 189 (65%) | 13 (81%) |
| *＞30* | 104 (35%) | 3 (19%) |
| **Education** |  |  |
| *High school or below* | 56 (19%) | 6 (38%) |
| *College* | 211 (72%) | 10 (62%) |
| *Master’s degree or above* | 26 (9%) | 0 (0%) |
| **Monthly Income (USD, $)** |  |  |
| *<225* | 23 (8%) | 1 (6%) |
| *225-450* | 16 (5%) | 3 (19%) |
| *451-750* | 69 (23%) | 5 (31%) |
| *751-1200* | 100 (34%) | 3 (19%) |
| *>1200* | 85 (29%) | 4 (25%) |
| **Sexual Orientation** |  |  |
| *Gay* | 212 (72%) | 9 (56%) |
| *Others*\| | 81 (28%) | 7 (44%) |
| **Marital Status** |  |  |
| *Single* | 246 (84%) | 15 (94%) |
| *Engaged or married* | 37 (13%) | 0 (0%) |
| *Separated or divorced* | 10 (3%) | 1 (6%) |
| Residence |  |  |
| *Guangdong province* | 201 (68%) | 10 (62%) |
| *Others* | 92 (32%) | 6 (38%) |

Data are presented as n (%). |Others include heterosexual, bisexual, and not sure.
