# Supplementary material for: Monetary incentives and peer referral in promoting secondary distribution of HIV self-testing among men who have sex with men in China: A randomized controlled trial
Source: PLoS Med. 2022 Feb 14;19(2):e1003928. doi: 10.1371/journal.pmed.1003928 (PMC8887971; doi:10.1371/journal.pmed.1003928)
Supplement: S2 Table — (DOCX) [file pmed.1003928.s002.docx]

**S2 Table. Summary of the total number of ordered kits and actual kits distribution by index participants in each arm, 2019-2020 (N=309)**

|  | **Control***  **N=102 (%)** | **SD-M†**  **N=103 (%)** | **SD-M-PR‡**  **N=104 (%)** |
| --- | --- | --- | --- |
| **Number of ordered kits** | |  |  |
| 1 | 40 (39) | 29 (28) | 35 (34) |
| 2 | 30 (29) | 29 (28) | 30 (29) |
| 3 | 17 (17) | 17 (17) | 12 (12) |
| 4 | 4 (4) | 3 (3) | 4 (4) |
| 5 ^a^ | 11 (11) | 25 (24) | 23 (22) |
| **Number of ordered peer-referral link** | |  |  |
| 1 | NA | NA | 68 (65) |
| **Number of actual kits distribution** | |  |  |
| 0 | 62 (61) | 54 (52) | 53 (51) |
| 1 | 31 (30) | 27 (26) | 22 (21) |
| 2 | 5 (5) | 4 (4) | 15 (14) |
| 3 | 1 (1) | 8 (8) | 2 (2) |
| 4 | 1 (1) | 8 (8) | 8 (8) |
| 5 | 2 (2) | 2 (2) | 4 (4) |
| >5 | NA | NA | 3 (3) |

*Control refers to a standard secondary distribution group. SD-M refers to secondary distribution with monetary incentives group. ‡SD-M-PR refers to secondary distribution with monetary incentives plus peer referral group. ^a^ The maximum number of ordered kits was the same (5) in each group. In the SD-M-PR group, index participants could request virtual peer-referral links and share links with alters who could use links to order kits which were not included in the number of ordered kits from index participants.
