# Supplementary material for: Monetary incentives and peer referral in promoting secondary distribution of HIV self-testing among men who have sex with men in China: A randomized controlled trial
Source: PLoS Med. 2022 Feb 14;19(2):e1003928. doi: 10.1371/journal.pmed.1003928 (PMC8887971; doi:10.1371/journal.pmed.1003928)
Supplement: S3 Table — (DOCX) [file pmed.1003928.s003.docx]

**S3 Table. Characteristics of the alters in China, 2019-2020 (N=269)**

|  | **Control***  **(n=58)** | **SD-M†**  **(n=101)** | **SD-M-PR‡**  **(n=110)** | **Overall**  **(n=269)** |
| --- | --- | --- | --- | --- |
| Missing data | 4 | 0 | 0 | 4 |
| **Age (years)** | 27·87 (6·96) | 27·61 (6·47) | 28·20 (7·61) | 27·91 (7·04) |
| ≤ 30 | 42 (78%) | 78 (77%) | 82 (75%) | 202 (76%) |
| ＞30 | 12 (22%) | 23 (23%) | 28 (25%) | 63 (24%) |
| Sex |  |  |  |  |
| Male | 54 (100%) | 101 (100%) | 110 (100%) | 269 (100%) |
| Gender |  |  |  |  |
| Male | 52 (96%) | 100 (99%) | 107 (97%) | 259 (98%) |
| Female | 1 (2%) | 0 (0%) | 2 (2%) | 3 (1%) |
| Not sure | 1 (2%) | 1 (1%) | 1 (1%) | 3 (1%) |
| **Education** |  |  |  |  |
| High school or below | 15 (28%) | 26 (26%) | 30 (30%) | 71 (27%) |
| College | 30 (56%) | 70 (69%) | 77 (76%) | 177 (67%) |
| Master’s degree or above | 9 (17%) | 5 (5%) | 3 (3%) | 17 (6%) |
| **Monthly Income (USD, $)** |  |  |  |  |
| <225 | 2 (4%) | 4 (4%) | 12 (11%) | 18 (7%) |
| 225-450 | 3 (6%) | 8 (8%) | 11 (10%) | 22 (8%) |
| 451-750 | 13 (24%) | 30 (30%) | 24 (22%) | 67 (25%) |
| 751-1200 | 21 (39%) | 31 (31%) | 47 (43%) | 99 (37%) |
| >1200 | 15 (28%) | 28 (28%) | 16 (15%) | 59 (22%) |
| **Sexual Orientation** |  |  |  |  |
| Gay | 37 (69%) | 74 (73%) | 70 (64%) | 181 (68%) |
| Others\| | 17 (31%) | 27 (27%) | 40 (36%) | 84 (32%) |
| **Sexual Orientation Disclosure¶** |  |  |  |  |
| Yes | 22 (41%) | 38 (38%) | 38 (35%) | 98 (37%) |
| No | 32 (59%) | 63 (62%) | 72 (65%) | 167 (63%) |
| **Marital Status** |  |  |  |  |
| Single | 44 (81%) | 80 (79%) | 87 (80%) | 211 (80%) |
| Engaged or married | 3 (6%) | 16 (16%) | 19 (17%) | 38 (35%) |
| Separated or divorced | 7 (13%) | 5 (5%) | 4 (4%) | 16 (6%) |
| **Condomless sex#** |  |  |  |  |
| Yes | 17 (31%) | 27 (27%) | 18 (16%) | 62 (24%) |
| No | 37 (69%) | 74 (73%) | 92 (84%) | 203 (77%) |
| **Relationship with index** |  |  |  |  |
| Same-sex relationship | 20 (37%) | 37 (37%) | 40 (36%) | 97 (37%) |
| Different-sex relationship | 2 (4%) | 2 (2%) | 5 (5%) | 9 (3%) |
| Same-sex friendship | 25 (46%) | 54 (53%) | 60 (55%) | 139 (52%) |
| Different-sex friendship | 2 (4%) | 1 (1%) | 1 (1%) | 4 (2%) |
| Family members | 1 (2%) | 1 (1%) | 3 (3%) | 5 (2%) |
| Others | 4 (7%) | 6 (6%) | 1 (1%) | 11 (4%) |
| Co-testing with index |  |  |  |  |
| Yes | 25 (46%) | 43 (43%) | 54 (49%) | 122 (46%) |
| No | 29 (54%) | 58 (57%) | 56 (51%) | 143 (54%) |

Age data are presented as mean (SD). Other data are presented as n (%). Responses of variables may not add up to the total due to missing values, and valid percentages were calculated *Control refers to standard secondary distribution group. †SD-M refers to secondary distribution with monetary incentives group. ‡SD-M-PR refers to secondary distribution with monetary incentives plus peer referral group. 110 alters in the SD-M-PR group are excluded alters who used kits ordered through peer-referral links. |Others include heterosexual, bisexual, and not sure. ¶Sexual Orientation Disclosure refers to whether an alter disclose their sexual orientation to people other than sexual partners, e.g. healthcare providers, friends, family members etc. #Condomless sex in the past months refers to whether an alter had condomless sex with another man in the past three months.
