# Supplementary material for: Monetary incentives and peer referral in promoting secondary distribution of HIV self-testing among men who have sex with men in China: A randomized controlled trial
Source: PLoS Med. 2022 Feb 14;19(2):e1003928. doi: 10.1371/journal.pmed.1003928 (PMC8887971; doi:10.1371/journal.pmed.1003928)
Supplement: S4 Table — (DOCX) [file pmed.1003928.s004.docx]

**S4 Table. Cost of the three arms of the trial by fixed and variable costs (USD, 2020)**

|  | **Control*** | **SD-M†** | **SD-M-PR‡** |
| --- | --- | --- | --- |
| ***Total cost*** | 5578·44 | 6219·23 | 7688·30 |
| ***Fixed cost***§ |  |  |  |
| Capital | 919·40 | 919·40 | 919·40 |
| Staff | 2598·35 | 2598·35 | 2598·35 |
| Consumables | 326·15 | 326·15 | 326·15 |
| ***Variable cost***\| |  |  |  |
| Staff | 207·41 | 264·61 | 324·80 |
| Consumables | 1527·14 | 2110·73 | 3519·61 |

*Control refers to a standard secondary distribution group. †SD-M refers to secondary distribution with monetary incentives group. ‡SD-M-PR refers to secondary distribution with monetary incentives plus peer referral group. §Fixed costs included capital costs (building rent, office equipment), staff time (wages of personnel using human capital approach), and consumables (condoms, lubricants given to all index MSM). |Variable costs included staff time (wages of personnel using human capital approach) and consumables (HIVST kits and monetary incentive). The full excel file with cost items is available in S2 Data.
