# Supplementary figures and images for: Monetary incentives and peer referral in promoting secondary distribution of HIV self-testing among men who have sex with men in China: A randomized controlled trial
Source: PLoS Med. 2022 Feb 14;19(2):e1003928. doi: 10.1371/journal.pmed.1003928 (PMC8887971; doi:10.1371/journal.pmed.1003928)

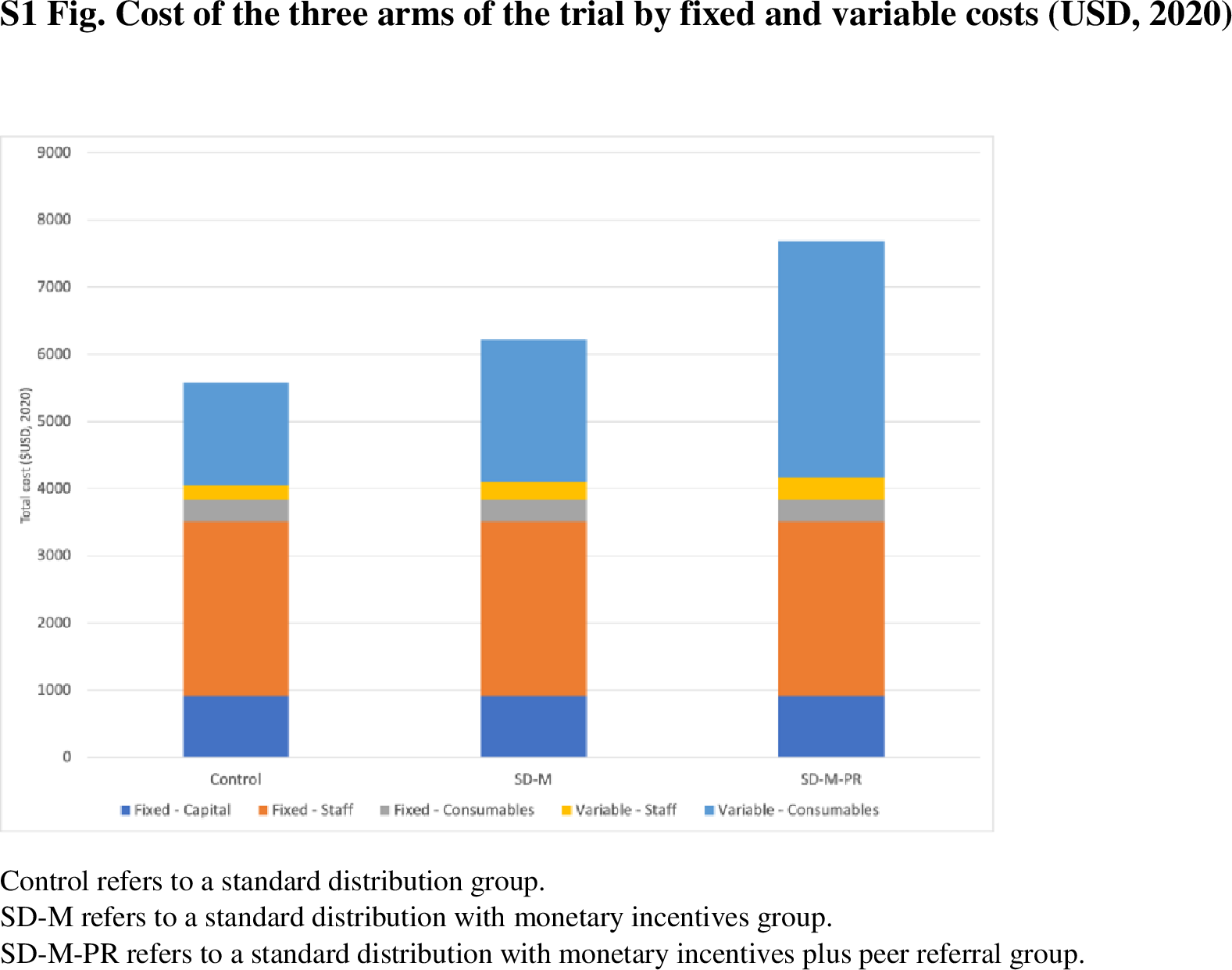

Supplement: S1 Fig — (TIF) [file pmed.1003928.s005.tif]

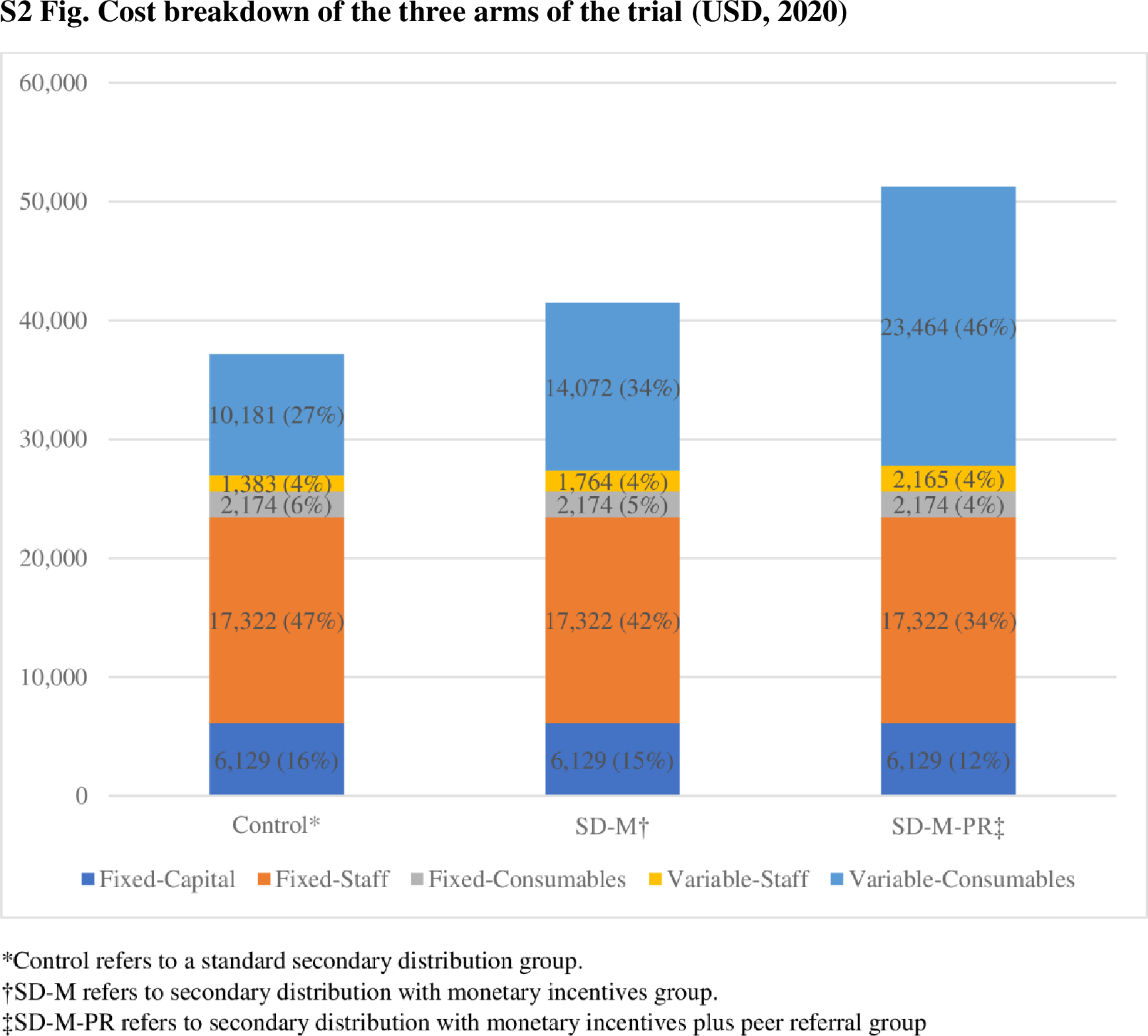

Supplement: S2 Fig — (TIF) [file pmed.1003928.s006.tif]
