# Supplementary material for: Monetary incentives and peer referral in promoting secondary distribution of HIV self-testing among men who have sex with men in China: A randomized controlled trial
Source: PLoS Med. 2022 Feb 14;19(2):e1003928. doi: 10.1371/journal.pmed.1003928 (PMC8887971; doi:10.1371/journal.pmed.1003928)
Supplement: S2 File — CHEERS, Consolidated Health Economic Evaluation Reporting Standards. (DOCX) [file pmed.1003928.s008.docx]

**S2 File. CHEERS Checklists**
