# Supplementary material for: Monetary incentives and peer referral in promoting secondary distribution of HIV self-testing among men who have sex with men in China: A randomized controlled trial
Source: PLoS Med. 2022 Feb 14;19(2):e1003928. doi: 10.1371/journal.pmed.1003928 (PMC8887971; doi:10.1371/journal.pmed.1003928)
Supplement: S3 File — (DOCX) [file pmed.1003928.s009.docx]

**S3 File. Study Protocol**

Lu Y, Ni Y, Li X, He X, Huang S, Zhou Y, et al. Monetary incentives and peer referral in promoting digital network-based secondary distribution of HIV self-testing among men who have sex with men in China: study protocol for a three-arm randomized controlled trial. BMC Public Health 2020;20:911. <https://doi.org/10.1186/s12889-020-09048-y>.

**Monetary Incentives and Peer Referral in Promoting Digital Network-Based Secondary Distribution of HIV Self-Testing among Men Who Have Sex with Men in China: Study Protocol for a Three-Arm Randomized Controlled Trial**

Ying Lu^1^**^§^**, Yuxin Ni^1^**^§^**, Xiaofeng Li^2^, Xi He^3^, Shanzi Huang^2^, Yi Zhou^2^, Wencan Dai^2^, Dan Wu^1,4^, Joseph D. Tucker^4^, Guangquan Shen^1^, Yongjie Sha^1^, Hongbo Jiang^5^, Liqun Huang^2^*, Weiming Tang^1^*

**^§^ Ying Lu and Yuxin Ni equally contribute to this study and are co-first authors.**

***Liqun Huang and Weiming Tang equally contribute to this study.**

**Address:** ^1^University of North Carolina Project-China, Guangzhou, China. ^2^Zhuhai Center for Diseases Control and Prevention, Zhuhai, China. ^3^Zhuhai Xutong Voluntary Services Center, Zhuhai, China. ^4^London School of Hygiene and Tropical Medicine, London, UK. ^5^Guangdong Pharmaceutical University, Guangzhou, China.

***Correspondence to**:

Weiming Tang, University of North Carolina Project-China, Guangzhou, China; Email: weiming_tang@med.unc.edu

**Abstract**

**Background**

Human immunodeficiency virus (HIV) testing is a crucial strategy for HIV prevention. HIV testing rates remain low among men who have sex with men (MSM) in China. Digital network-based secondary distribution is considered as an effective model to enhance HIV self-testing (HIVST) among key populations. Digital platforms provide opportunities for testers to apply for HIVST kits by themselves, and secondary distribution allows them to apply for multiple kits to deliver to their sexual partners or members within their social network. We describe a three-arm randomized controlled trial to examine the effect of monetary incentives and peer referral in promoting digital network-based secondary distribution of HIVST among MSM in China.

**Methods**

300 MSM in China will be enrolled through a digital platform for data collection. The eligibility criteria include being biological male, 18 years of age or over, ever having had sex with another man, being able to apply for kits via the online platform, and being willing to provide personal telephone number for follow-up. Eligible participants will be randomly allocated into one of the three arms: standard secondary distribution arm, secondary distribution with monetary incentives arm, and secondary distribution with monetary incentives plus peer referral arm. Participants (defined as “index”) will distribute actual HIV self-test kits to members within their social network (defined as “alter”) or share referral links to encourage alters to apply HIV self-test kits by themselves. All index participants will be requested to complete a baseline survey and a 3-month follow-up survey. Both indexes and alters will complete a survey upon returning the results by taking a photo of the used kits with the unique identification number.

**Discussion**

HIV testing rates remain suboptimal among MSM in China. Innovative interventions are needed to further expand the uptake of HIV testing among key populations. The findings of the trial can provide scientific evidence and experience on promoting secondary distribution of HIVST to reach key populations who have not yet been covered by existing testing services.

**Trial registration**

The study was registered in the Chinese Clinical Trial Registry (ChiCTR1900025433) on 26, August 2019, <http://www.chictr.org.cn/showproj.aspx?proj=42001>. Prospectively registered.

**Keywords**

HIV self-testing; monetary incentives; men who have sex with men; peer referral; secondary distribution

**Background**

**Background and rationale**

Men who have sex with men (MSM) are one of the key populations affected by human immunodeficiency virus (HIV).(1) Compared to general populations, the risk of acquiring HIV is 22 times higher in MSM.(2) In China, the HIV infection rate among MSM was 6.9% by 2018.(3) Additionally, a large-scale systematic analysis illustrated that prevalence of HIV among MSM in China increased substantially from 2001 to 2018.(4)

HIV testing is considered as a significant stage of the HIV care continuum(5) and the Treat All strategy,(6) because serostatus awareness can link patients to timely treatment and prevent wider transmission of HIV.(7) Thus, expanding HIV testing is crucial for HIV prevention and treatment. However, conventional HIV test services like healthcare facility-based tests fail to reach a wider hidden group of people, mainly due to the barriers including the stigma of HIV testing, the lack of confidentiality and privacy, low trust towards healthcare institutions, and inconvenience.(8–11) To increase HIV testing among people who do not know their HIV status, the World Health Organization (WHO) recommends HIV self-testing (HIVST) as an empowering and innovative way to reach those who have limited access to HIV testing and those who are at high risk of HIV infection.(12) With HIVST, individuals can decide where and when to test while ensuring efficiency, privacy, and confidentiality. HIVST may be an effective alternative for those who do not regularly attend healthcare facilities, which may also be a promising approach to increase the uptake of HIV testing in key populations such as MSM.(13,14)

Digital network-based secondary distribution of HIVST could be an effective strategy for promoting HIVST. This is a strategy that individuals (defined as indexes) to apply for multiple HIVST kits and distribute them to their sexual partners or other members (defined as alters) within their social network.(14,15) A cohort study conducted in Kenya has proven the feasibility and acceptability of secondary distribution of HIVST among female sex workers.(14) One observational study in China also indicated that secondary distribution of HIVST successfully reached people who were not covered by traditional testing services and promoted case identification.(16)

Digital health is also considered to be an innovative strategy to deal with traditional health challenges, including challenges for HIV prevention.(17) For example, digital health has been used to promote safe sex, condom use, and awareness of HIV or sexually transmitted diseases among key populations.(18,19) For MSM, social networking or dating apps are widely used, especially among young MSM, which provides a unique opportunity of leveraging digital health in improving health services among them. Combining digital health and social network-based strategy for HIV testing (i.e., secondary distribution) promotion can help surpass traditional barriers and reach more people who have never been reached by the facility-based services and increase case finding.

In this study, we intend to examine two modified secondary distribution approaches for HIVST through a three-arm randomized controlled trial in Zhuhai, China. The main purpose of this study is to compare the effectiveness of two modified secondary distribution approaches (monetary incentives, and monetary incentives plus peer referral) with the traditional secondary distribution approach, in order to determine whether these two approaches can increase the uptake of HIVST among MSM, especially the first-time testers among alters.

**Objectives**

Our trial aims to enable more Chinese MSM to receive HIV self-testing, reach more first-time HIV testing alters, and identify more people with an HIV-positive (reactive) result by implementing the photo-verified HIV self-testing method under different scenarios, i.e., standard secondary distribution, secondary distribution with monetary incentives and secondary distribution with monetary incentives plus peer-referral links.

**Hypotheses**

Hypothesis 1: Compared with standard secondary distribution, secondary distribution with monetary incentives will promote index MSM to distribute more HIVST kits to people within their social network.

Hypothesis 2: Compared with standard secondary distribution, secondary distribution with monetary incentives plus peer referral will promote index MSM to distribute more HIVST kits to people within their social network.

Hypothesis 3: Compared with monetary alone secondary distribution, secondary distribution with monetary incentives plus peer referral will promote index MSM to distribute more HIVST kits to people within their social network.

**Trial design**

This is a three-arm randomized controlled trial among Chinese MSM. Enrolled indexes will be randomly assigned to one of the three groups: standard secondary distribution, secondary distribution with monetary incentives arm, and secondary distribution with monetary incentives plus peer referral. Further, indexes will be asked to complete a baseline survey at the beginning of the trial, and a three-month follow-up survey after their HIVST kits applications. A flowchart of the trial is shown in Figure. 1.

**Methods/Design**

**Study setting and recruitment**

This trial, conducted in Zhuhai, is a collaboration among the Social Entrepreneurship to Spur Health (SESH) research team, Zhuhai Center of Diseases Control (CDC), and Zhuhai Xutong Voluntary Services Center (hereafter, Xutong). Zhuhai, located in Southern China, has approximately 17,000 MSM with an HIV prevalence rate of 7%.(12) Xutong is a local gay community-based organization (CBO) founded in 2015 and has developed a digital network-based platform for individuals (all-over time) to apply for free HIV/syphilis self-testing kits provided by Zhuhai CDC. With Xutong’s social impact within the gay community, its volunteers will help with study recruitment, and their online platform will be used to support our intervention implementation.

Study recruitment advertisement will be posted via Xutong’s official account on WeChat, a popular social site in China similar to Facebook and Twitter. The recruitment information will also be advertised on BlueD, the largest social network app within the gay community in China. Potential participants can join the trial via the study ads and sign up for Xutong’s online platform.

**Eligibility criteria**

An eligible index is required to meet the following criteria: 1) Chinese born biologically male whose age is 18 years old or older; 2) ever had sex with another man; 3) willing to self-apply HIVST kits via Xutong’s digital platform; 4) willing to provide personal contact information for future follow-up. All participants will need to sign an informed consent electronically before the study.

**Allocation**

Randomization will be completed by a computer-generated program with a 1:1:1 allocation ratio. A consented participant will be allocated to one of three study groups, i.e., standard secondary distribution group, secondary distribution with monetary incentives group, and secondary distribution with monetary incentives plus peer referral group.

**Interventions**

Standard secondary distribution arm/ Control arm: Index MSM in this arm will be eligible to apply for up to 5 HIVST kits based on personal needs, and a 100 RMB (≈15 USD) deposit will be charged for each kit. The charged deposit will be refunded to the index if anyone (an index himself or alters to whom he distributed) return his/her testing result. In addition, the participants will need to provide contact information for kits shipping. All kits shipped to a participant will be packed with instructions in an unmarked box to protect privacy. Each kit will be assigned with an identical confirmation code for future distribution tracking, and a unique “ST” number for returned results tracking. After receiving kits, an index can choose to use the kits for themselves or distribute additional kits to alters such as sexual partners or friends. Self-testing kit users can photograph and upload their results to the online platform anonymously by scanning the QR code attached on each kit box. Figure 2 shows the HIVST reporting QR code for testers to return results. Further, alters will be asked to fill out a survey regarding their experience of and attitude toward HIVST. After the survey is completed, the deposit will be returned to the matched index by tracking the confirmation code.

Secondary distribution with monetary incentives (SD/monetary incentives) arm: Index MSM in this arm will follow the same application process as in the control arm. Differently, a fixed 20 RMB (≈ 3 USD), designed as the financial incentives, will be offered to all self-testers who report their results. When an alter returns his/her results, his/her matched index MSM will also receive an extra 20 RMB as incentives.

Secondary distribution with monetary incentives plus peer referral (SD/monetary incentives plus peer referral) arm: Index MSM in this arm will first receive the same intervention as the participants received in the monetary incentives arm. In addition, apart from applying for up to five self-testing kits, each index MSM in this arm will obtain a unique referral link, which can be shared with up to 5 individuals within their social network to apply for kits, and each alter can apply for only one kit through the link. Similarly, indexes will be given 20 RMB for each matched alter who returns the result (whether through peer referral or direct distribution). All testers will receive a monetary incentive of 20 RMB once they complete uploading their results.

**Follow-up**

The follow-up survey will be administered three months after indexes apply for HIVST kits. This survey focuses on distribution history, the relationship between indexes and alters, and indexes’ risky sexual behavior. The results from follow-up survey will be compared with baseline survey results to investigate whether the index MSM has changed their behavior after HIVST. Xutong’s volunteers will check returned self-test results. If there is any positive (reactive) result returned, volunteers will contact testers accordingly, and refer them to the local CDC to do confirmation tests. Moreover, reactive testers will be encouraged to distribute HIVST kits to their sexual partners or provide partners’ contact information to volunteers for offering free testing service.

**Outcomes**

The primary outcomes of this trial consist of three parts: 1) Mean number of motivated alters who have photo-verified self-testing per index in each arm; 2) Proportion of first-time HIV testing among alters in each arm; 3) Proportion of alters with a positive HIV testing result in each arm.

The secondary outcomes of this study consist of three parts 1) Risky sexual behavior among indexes and alters in each arm; 2) Adverse events reported during secondary distribution among indexes and alters in each arm; 3) Attitude towards and past experiences of HIVST and sexual behavior among alters in each arm.

**Measures**

In general, we aim to examine the effectiveness of our modified secondary distribution models. Primary outcomes of this trial are the number of alters, first-time testing alters, and HIV-positive alters in each arm, therefore, all testers’ information will be specifically clarified and recorded. All data will be collected through JINSHUJU, a secure online platform where participants can apply for HIVST kits, report testing results, and complete the baseline and follow-up questionnaires. All data collected from participants will be examined and deduplicated according to phone number and IP.

In the baseline survey, we will collect the socio-demographic characteristics, sexual orientation, sexual behavior, HIV testing history, and social network. When alters return the results, they will also complete a questionnaire online. In this survey, except for basic information on socio-demographic characteristics, sexual orientation, sexual behavior, HIV testing history, and social network will be collected. We will also investigate the attitudes and experiences towards self-test. The follow-up survey for indexes will take place three months after the application completed, which focuses on experiences and attitudes on using and distributing self-test kits. The survey mainly inquires about the occurrence of intimate partner violence (IPV) or other adverse events, the relationship between the indexes and the alters, whether indexes have tested together with the alters, and whether indexes have guided the alters on how to perform HIVST.

Specifically, in our surveys, socio-demographic characteristics include indexes’ age, sex, marital status, the highest level of education completed, and monthly income level. Sexual behavior within three months includes previous sex with males and females, role during sex with males, condom use, number of sex partners, and drug use. HIV testing history includes health care facility-based and online HIV testing experience. Social network collects community engagements,(7) community connectedness,(20) identity fusion,(21) and social cohesion.(22)

All blood samples will be analyzed using *SD Bioline* HIV/Syphilis duo test kits (*SD Bioline Company*, South Korea). Participants will collect fingertip blood samples by themselves according to the instruction. The trained staff of the CDC or CBO will check and read the photos of result and record them in JINSHUJU. The results are subject to the reading of the staff.

**Missing data plan**

Participants will be involved at one to three stages: the baseline questionnaire, results return, and follow-up survey. However, there might be data missing in the primary and secondary outcomes. If there is <20% of participants missing in the follow-up, a complete-case approach will be applied. If there is ≥ 20% of participants missing in the follow-up, we will investigate the missingness mechanism and use suitable imputation.

**Sample size**

According to preliminary study results, on average, the number of alters motivated by an index man was 0.65 through standard secondary distribution, 1.0 through SD/monetary incentives intervention, and 1.4 through SD/monetary incentives plus peer referral intervention. We assumed that the variances of the three groups were equal with the same standard deviation of 0.5 (preliminary data, unpublished results). Further, we estimated an effective sample size of 300 participants (100 in each group), with a power of 0.90, an alpha of 0.05, and a lost to follow-up rate of 0.20.

**Data analysis**

All statistical analyses will follow the intention-to-treat principle. Missing data will be handled by multiple imputations. Categorical variables from the baseline survey will be aggregated in frequency distributions, and numerical variables will be summarized in mean and standard deviation.

Primary Analysis: We will first calculate primary outcomes in each arm, i.e., mean number of alters that each index recruited, proportion of first-time HIV testers, and proportion of alter testers with an HIV-positive result. Secondly, we will compare calculated means using two-sample t-test, and proportions using chi-square between SD/monetary incentives arm and control arm, SD/monetary incentives plus peer referral arm and control arm, and also between two intervention arms.

Secondary Analysis: Results of the follow-up survey for indexes and the survey for alters will be used for measuring secondary outcomes, i.e. risky sexual behavior, adverse events during secondary distribution, and experience and attitude towards HIV testing. High-risk sexual behavior, defined as unprotected sex, substance use, or multiple sexual partners, are determined from survey questions such as “How often do you wear a condom during anal sex?”, “In the past three months, how many sexual partners did you have?”, “In the past three months, have you used drugs before sex?”, etc. Adverse events or IPV that happened on alters during distribution are determined from the question “Have you experienced any of the following IPV when received the HIVST kit from the index?”. Alters’ experience and perception of HIVST are determined from total 20 question items, e.g. “How difficult do you feel about completing HIV self-testing?”, “How many men did you have anal sex with after HIV self-testing?”, “Which testing do you prefer, facility-based testing or HIV self-testing?”, etc. We will use chi-square tests to compare each outcome of interests between SD/monetary incentives group and control group, SD/monetary incentives and peer referral group and control group, and also between two intervention arms.

**Discussion**

Despite global HIV control programs, HIV persists as a major public health threat among key populations such as MSM.(12) Therefore, the screening of HIV in MSM plays a key role in furthering prevention and control. HIVST was considered to be an alternative strategy to promote HIV testing. Digital network-based secondary distribution takes advantage of digital network, which can be a promising approach to enhance HIVST. We apply innovative strategies on the basis of secondary distribution model, adding monetary incentives and peer referral to explore a more effective secondary distribution model and promote HIVST to wider populations.

However, it is necessary to consider several limitations of this trial. First, due to the digital network strategy, access to this HIVST service is limited so that the recruitment might overlook individuals who cannot access online social tools. However, the mobilization and promotion from the local CBO Xutong, as well as the face-to-face distribution initiated by index MSM can somehow resolve the problem. Second, in the online surveys, behaviors of participants are self-reported, which may increase the possibility of social desirability bias. It can lead to the Hawthorne effect that participants may have deviations in behavior reporting because of the awareness of the trial. However, the form of the online questionnaire and limiting collection of identifiers can reduce bias. Third, from the perspective of implementation and promotion, each participant can only apply once due to the design of the trial, while there may be participants who have the habit of regular testing and request to apply for HIVST kits multiple times. While in this project we cannot satisfy such demand, the future implementation model can expand access to multiple applications.

This study generates a social innovation and policy implication that expands and improves the public service of HIV prevention and control, with the use of social network strategies. From the perspective of digital health, digital network-based secondary distribution of HIVST is an innovative delivery approach that can reach hidden MSM. Beyond the geographical limitation, some MSM, especially those in remote conservative areas, have limited access to facility-based services, while digital platforms might motivate them to perform HIV testing. Furthermore, by taking advantage of digital network-based distribution, we aim to reduce fear of stigma associated with conventional HIV testing services in healthcare facilities. Social network strategies, especially peer referral, taps into self-identity with a community and trust to provide services more effectively. Empowering vulnerable individuals within a community by offering essential resources has both research and policy implementations for expansion of HIV services in other hard-to-reach populations. Upon completion of the study, we will provide the community with practical digital network-based HIVST interventions and scientific evidence on the feasibility and acceptability of the secondary distribution approaches. In addition, practical experience and knowledge gained from conducting the interventions can be considered and applied to further trials in enhancing HIV testing. If successful, the strategy has the potential to be implemented in similar regions.

**Abbreviations**

CBO: Community-Based Organizations

CDC: Center for Disease Control and Prevention

HIV: Human Immunodeficiency Virus

HIVST: HIV Self-Testing

IPV: Intimate Partner Violence

MSM: Men Who Have Sex with Men

SD: Secondary Distribution

SESH: Social Entrepreneurship to Spur Health Group

WHO: World Health Organization

**Trial status**

The study timeline was designed from 01, September 2019 to 31, May 2020. At the time of writing this draft protocol, study enrollment and data collection are ongoing. Due to COVID-19 pandemic, study recruitment expects to be delayed till 31, September 2020, and the follow-up will be finished by 31 December 2020. Thus, we have further updated the trial registration status, and updated the study period as 01, September 2019 to 31, December 2020. Ethics approval will be renewed annually. Statistical analysis has not begun. The trial protocol conforms to the Standard Protocol Items: Recommendation for Interventional Trials (SPIRIT) 2013 statement.

**Declarations**

**Ethics approval and consent to participate**

Ethical review of biomedical research has been obtained from the Ethics Committee of Zhuhai Center for Disease Control and Prevention prior to study enrollment. All participants will be provided online consents and sign it electronically prior to taking part in the study.

**Consent for publication**

Not applicable

**Availability of data and materials**

Data sharing is not applicable to this article as no datasets were generated or analyzed during the current study.

**Competing interests**

The authors declare that they have no competing interests.

**Funding**

This study received support from internal institute funding of Zhuhai Center for Disease Prevention and Control, and the National Institutes of Health (NIMH 1R34MH119963-01). The funding source had no role in the process of study design, data collection, and analysis, decision to publish, or preparation of the manuscript.

**Acknowledgements**

We appreciate the contributions from all study participants, CBO volunteers, and staff from Zhuhai Center for Disease Control and Prevention, Zhuhai Xutong Voluntary Services Center, and Social Entrepreneurship to Spur Health Group.

**Authors’ Contribution**

YL and YN drafted and finalized the paper with inputs from WT, DW and GS. JT provided critical revision of the paper. YZ, DW, LH, and WT conceived the study. XH, XL, YS and SH assisted with recruitments. WT, YZ, DW, JT, LH, WD, GS and HJ provided oversight. WD and HJ made insightful contributions to the study conception. All authors read and authorized the final version.

**References**

1. Beyrer C, Baral SD, Griensven F van, Goodreau SM, Chariyalertsak S, Wirtz AL, et al. Global epidemiology of HIV infection in men who have sex with men. The Lancet. 2012 Jul 28;380(9839):367–77.

2. UNAIDS. Global HIV & AIDS statistics — 2019 fact sheet [Internet]. 2019 [cited 2020 Mar 2]. Available from: https://www.unaids.org/en/resources/fact-sheet

3. UNAIDS. KEY POPULATIONS ATLAS [Internet]. [cited 2020 Apr 20]. Available from: http://kpatlas.unaids.org/dashboard

4. Dong M-J, Peng B, Liu Z-F, Ye Q, Liu H, Lu X-L, et al. The prevalence of HIV among MSM in China: a large-scale systematic analysis. BMC Infect Dis. 2019 Nov 27;19(1):1000.

5. Kilmarx PH, Mutasa-Apollo T. Patching a leaky pipe: the cascade of HIV care. Curr Opin HIV AIDS. 2013 Jan;8(1):59–64.

6. 90-90-90: An ambitious treatment target to help end the AIDS epidemic. Geneva: UNAIDS; 2014 p. 40.

7. Zhang TP, Liu C, Han L, Tang W, Mao J, Wong T, et al. Community engagement in sexual health and uptake of HIV testing and syphilis testing among MSM in China: a cross-sectional online survey. J Int AIDS Soc. 2017 03;20(1):21372.

8. Tucker JD, Muessig KE, Cui R, Bien CH, Lo EJ, Lee R, et al. Organizational characteristics of HIV/syphilis testing services for men who have sex with men in South China: a social entrepreneurship analysis and implications for creating sustainable service models. BMC Infect Dis. 2014 Nov 25; 14:601.

9. Song Y, Li X, Zhang L, Fang X, Lin X, Liu Y, et al. HIV-testing behavior among young migrant men who have sex with men (MSM) in Beijing, China. AIDS Care. 2011 Feb;23(2):179–86.

10. Krause J, Subklew-Sehume F, Kenyon C, Colebunders R. Acceptability of HIV self-testing: a systematic literature review. BMC Public Health. 2013 Aug 8; 13:735.

11. Tucker JD, Bien CH, Peeling RW. Point-of-care testing for sexually transmitted infections: recent advances and implications for disease control. Curr Opin Infect Dis. 2013 Feb;26(1):73–9.

12. WHO. Status of HIV self-testing in national policies [Internet]. WHO. World Health Organization; 2019 [cited 2020 Mar 3]. Available from: http://www.who.int/hiv/topics/self-testing/en/

13. Myers JE, El-Sadr WM, Zerbe A, Branson BM. Rapid HIV self-testing: long in coming but opportunities beckon. AIDS Lond Engl. 2013 Jul 17;27(11):1687–95.

14. Thirumurthy H, Masters SH, Mavedzenge SN, Maman S, Omanga E, Agot K. Promoting male partner testing and safer sexual decision-making through secondary distribution of HIV self-tests by HIV-uninfected female sex workers and women receiving antenatal and postpartum care in Kenya: a cohort study. Lancet HIV. 2016 Jun;3(6): e266–74.

15. Masters SH, Agot K, Obonyo B, Napierala Mavedzenge S, Maman S, Thirumurthy H. Promoting Partner Testing and Couples Testing through Secondary Distribution of HIV Self-Tests: A Randomized Clinical Trial. PLoS Med [Internet]. 2016 Nov 8 [cited 2020 May 1];13(11). Available from: https://www.ncbi.nlm.nih.gov/pmc/articles/PMC5100966/

16. Dan Wu. Social-media based secondary distribution of HIV self-testing among Chinese men who have sex with men: A pilot implementation program assessment [Internet]. IAS2019; 2019 Jul 24 [cited 2020 Apr 27]. Available from: http://programme.ias2019.org/Abstract/Abstract/3026

17. Cao B, Bao H, Oppong E, Feng S, Smith KM, Tucker JD, et al. Digital health for sexually transmitted infection and HIV services: a global scoping review. Curr Opin Infect Dis. 2020 Feb;33(1):44–50.

18. Ko N-Y, Hsieh C-H, Wang M-C, Lee C, Chen C-L, Chung A-C, et al. Effects of Internet popular opinion leaders (iPOL) among Internet-using men who have sex with men. J Med Internet Res. 2013 Feb 25;15(2): e40.

19. Kelly JA. Popular opinion leaders and HIV prevention peer education: resolving discrepant findings, and implications for the development of effective community programmes. AIDS Care. 2004 Feb;16(2):139–50.

20. Frost DM, Meyer IH. Measuring Community Connectedness among Diverse Sexual Minority Populations. J Sex Res. 2012 Jan;49(1):36–49.

21. Swann Jr. WB, Gómez Á, Seyle DC, Morales JF, Huici C. Identity fusion: The interplay of personal and social identities in extreme group behavior. J Pers Soc Psychol. 2009;96(5):995–1011.

22. Grover E, Grosso A, Ketende S, Kennedy C, Fonner V, Adams D, et al. Social cohesion, social participation and HIV testing among men who have sex with men in Swaziland. AIDS Care. 2016;28(6):795–804.
