# Supplementary material for: Monetary incentives and peer referral in promoting secondary distribution of HIV self-testing among men who have sex with men in China: A randomized controlled trial
Source: PLoS Med. 2022 Feb 14;19(2):e1003928. doi: 10.1371/journal.pmed.1003928 (PMC8887971; doi:10.1371/journal.pmed.1003928)
Supplement: S4 File — (DOCX) [file pmed.1003928.s010.docx]

S4 File. Study protocol amendments

## Protocol Amendments

Protocol Title: Monetary incentives and peer referral in promoting the digital network-based secondary distribution of HIV self-testing among men who have sex with men in China: study protocol for a three-arm randomized controlled trial.

Revisions to V1.0: 12 Jun 2020 (Original protocol)

Date: V2.0: 29 Sep 2021 (Amendment 02)

| Change | Rationale | Affected Protocol Sections |
| --- | --- | --- |
| Primary outcomes were changed to 1) number of unique tested alters motivated by each index and 2) number of newly tested alters motivated by each index for each arm. Secondary outcomes were changed to 1) number of alters who tested positive for HIV recruited by each index; 2) evaluation of intervention effects for the mean number of alters motivated by each index in subgroups defined by age, residence, sexual orientation, sexual behavior, and previous testing experience; and 3) the cost per person tested and cost per HIV diagnosed for each arm. | 1. To make the overall results reporting more logical and clearer   In our study, the first primary outcome was measured in the number of motivated alters who have self-tested each arm, which was not changed. Rather, we replaced the second primary outcome with the “number of newly tested alters motivated by each index for each arm.” This change can make the two primary outcomes consistent with each other, which both are measured in number. By making this consistency, we believe the overall results reporting is more logical, clearer, and easier to understand, as you can see in table 2 in our manuscript. Even if we have changed this, we continued to report the proportion of newly tested alters in the manuscript: “Of the unique alters in each group (control group: 58; SD-M: 101; SD-M-PR: 185), 28% (16/58) of alters in the control group were newly tested, compared to 42% (42/101) of alters in the SD-M, and 32% (59/185) of alters in the SD-M-PR (Figure 2)”.   1. To align with the sample size calculation   Our sample size calculation was based on the number of motivated alters who have uploaded photo-verified self-testing results per index in each arm instead of proportion. This change can make the outcomes align with the sample size calculation.   1. To make the results easier to be understood by the implementers   This study extended our pilot study by adding the two new interventions to motivate more individuals for testing and increasing coverage. The main purpose is to encourage more people, especially more new testers to conduct HIVST and the study was mainly implemented by the local gay-led CBO in Zhuhai. Considering more from the implementation perspective, we therefore reported the outcomes in number and calculated “the cost per person tested and cost per HIV diagnosed” additionally, which can make the outcomes easier to be understood by the wider community, especially the volunteers and staff of the CBO. This change will facilitate the dissemination of the study findings and would be useful for further scale-up of the interventions. | Outcome <Page 5> |
| Methods of outcome analyses were changed to use zero-inflated negative binomial regression to estimate the incidence rate ratio and 95% confidence interval. For the additional cost evaluation, we used microcosting to estimate the total cost of each trial arm. | In the original protocol, we mentioned that “We will compare calculated means using a two-sample t-test, and proportions using chi-square between SD/monetary incentives arm and control arm, SD/monetary incentives plus peer referral arm and control arm, and also between two intervention arms.” In the original study plan, we did not take the distribution of the outcomes into consideration. During the data analysis stage, we used Shapiro-Wilk's tests to test the normality of the primary outcomes, and they were not following the normal distribution. In this way, if we continue to use a two-sample t-test, the results will be biased. In addition, our outcome variables were all counts, and therefore, we applied negative binomial regression for constructing incidence rate ratios and two-sided 95% confidence intervals (CI).  Negative binomial regression is more suitable as the variance of the count number is larger than the mean value thus, assumptions of Poisson regression are not met. More specifically, we have tried Zero-inflated negative binomial regression as the count number data exhibits some dispersion and excess zeros.  We then illustrate the reason why we directly use number (e.g., alters with HIV-reactive results identified by each index in Control/SD-M/SD-M-PR) instead of the proportion testing (e.g., the proportion of indexes who distributed at least one kit to HIV-reactive alters in Control/SD-M/SD-M-PR). When we do proportion testing with risk difference (95% CI) analysis, it’s based on the assumption of Normal Approximation to Binomial Distribution, which strictly requires that np>5 and n(1-p)>5 both hold [Lumley, Thomas, et al. "The importance of the normality assumption in large public health data sets." Annual review of public health 23.1 (2002): 151-169. ]. However, our sample data didn’t meet such requirements. Thus using proportion difference testing methods could be not reliable here.  As a result, a zero-inflated negative regression method to analyze study outcomes is adopted in our manuscript. | Primary analysis, secondary analysis  <Page 6> |
