# Supplementary material for: Monetary incentives and peer referral in promoting secondary distribution of HIV self-testing among men who have sex with men in China: A randomized controlled trial
Source: PLoS Med. 2022 Feb 14;19(2):e1003928. doi: 10.1371/journal.pmed.1003928 (PMC8887971; doi:10.1371/journal.pmed.1003928)
Supplement: Attachment — Submitted filename: Secondary HIVST distribution among MSM.docx [file pmed.1003928.s013.docx]

Abstract

The paper describes a trial of two HIV self-test secondary distribution strategies among men having sex with men in China: incentives and online peer referral. It is an important study given the need to improve uptake of testing among key populations. There are however methodological weaknesses that call for caution in the interpretation of the findings:

**Major comments**

1. Deposit refunds were only given upon upload of test results. This could have forced index participants to over test so they could get reimbursed – there were 150 tests from 102 control participants, showing unnecessary testing and potential wastefulness of the intervention. Related to this, in lines 384-389, it does not seem convincing that the restriction on number of kits ordered was actually a limitation as it seems people ordered more than they could distribute
2. In the peer referral arm an index could distribute to/refer more people (ten) compared to five in the other arms. This makes results on mean numbers of testers incomparable with the other arms
3. There were baseline differences by arm in participant characteristics. Please comment on potential for confounding

**Minor comments**

**Abstract**

4. Consider the rewording the definition of alters because in the abstract it sounds like an alter is the whole network/group, rather than one individual
5. Although it is clear in the manuscript, in the abstract it is unclear where self-test results were uploaded (line 16)
6. From the abstract it is not immediately clear what is being referred to as the digital approach

**Manuscript**

7. Page 13 lines 81-82: it is not clear what “may crowd them out” means
8. Line 158 – not clear whether the index received an additional 20rmb to the one described in line 157
9. Line 182 – Syphilis comes from nowhere without having been described in the methods nor any of the earlier sections. How and where were syphilis tests done?
