# Supplementary material for: Monetary incentives and peer referral in promoting secondary distribution of HIV self-testing among men who have sex with men in China: A randomized controlled trial
Source: PLoS Med. 2022 Feb 14;19(2):e1003928. doi: 10.1371/journal.pmed.1003928 (PMC8887971; doi:10.1371/journal.pmed.1003928)
Supplement: Attachment — Submitted filename: Response letter.29Sept_final_updated.docx [file pmed.1003928.s015.docx]

Dear Dr. Davidson,

We are grateful for the helpful comments. We have used the comments to improve the manuscript. Please find our point-to-point responses, a marked-up copy of the revised manuscript, and a clean copy of the revised manuscript.

Weiming Tang, Ying Lu, and Yuxin Ni on behalf of the team

——————————————————————————————
**Part 1, Response to the comments from the Editors**

**Comment 1:** “In the interest of brevity, please consider shortening your title to ‘Monetary Incentives and Peer Referral in Promoting Secondary Distribution of HIV Self-Testing among Men Who Have Sex with Men in China: A Randomized Controlled Trial.”

**Response**: Thanks for the suggestion. We have shortened the title to “Monetary Incentives and Peer Referral in Promoting Secondary Distribution of HIV Self-Testing among Men Who Have Sex with Men in China: A Randomized Controlled Trial.”

**Comment 2**: “The Data Availability Statement (DAS) requires revision - note that a study author cannot be the contact person for the data. Please direct data requests to a non-author institutional point of contact, such as a data access or ethics committee.”

**Response:** We have made the de-identified data available and revised the Data Availability Statement to: “All relevant data are within the paper and its Supporting Information files.” We have included the data as a supplement to the submission.

**Comment 3:** “Line 37: Please update to ‘Conclusions’”

**Response:** We have updated to “Conclusions.”

**Comment 4:** "Please trim the ‘Methods and Findings’ section of your abstract – the section should be a single paragraph containing the following:

* The study design, population and setting, number of participants, exact dates during which the study took place, length of follow up, definition of intervention and control states, who was blinded to the group allocation, and main outcome measures.

* Please also provide the number in each group, state that analysis was intention to treat, quantify the main results (with 95% CIs and p values), and include the important dependent variables that are adjusted for in the analyses. Please also include a summary of adverse events if these were assessed in the study.

* In the last sentence of the Abstract Methods and Findings section, please describe the main limitation(s) of the study's methodology.”

**Response:** Thanks for the guidance. The abstract has been revised as per request. A revised version of “Methods and Findings” is provided below for your reference.

“Between October 21, 2019, and September 14, 2020, a three-arm randomized controlled, single-blinded (statistician) trial was conducted online among 309 individuals (defined as index) who were assigned male at birth, aged 18 years or older, ever had male-to-male sex, willing to order HIVST kits online, and consented to take surveys online. Index participants were randomly assigned into one of the three arms: 1) standard secondary distribution (control) group (n=102), 2) secondary distribution with monetary incentives (SD-M) group(n=103), and 3) secondary distribution with monetary incentives plus peer referral (SD-M-PR) group (n=104). Index participants in three groups were encouraged to order HIVST kits online and distribute them to members within their social networks. Members who received kits directly from index MSM or through peer referral links from index MSM were defined as alters. Index participants in the two intervention groups could receive a fixed incentive ($3 USD) online for the verified test result uploaded to the digital platform by each unique alter. Index participants in the SD-M-PR group could additionally have a personalized peer referral link for sharing with alters to order kits online. All index participants were assigned an online 3-month follow-up survey after their kit ordering. The primary outcomes were measured by zero-inflated negative binomial regression. We also conducted an economic evaluation using microcosting from a health provider perspective with a 3-month time horizon. Results indicated that index participants in intervention arms were more likely to motivate unique tested alters (SD-M, IRR = 2.98, 95% CI= 1.82 – 4.89; SD-M-PR, IRR = 3.26, 95% CI = 2.29 – 4.63), and newly-tested alters to conduct HIVST (SD-M, IRR = 4.22, 95% CI= 1.93 – 9.23; SD-M-PR, IRR = 3.49, 95% CI = 1.92 – 6.37). The total costs were $19,485·97 for 794 testers, including 450 index participants and 344 alter testers. Overall, the average cost per tester was $24·54, and the average cost per alter tester was $56·65. Monetary incentives alone (SD-M) were more cost-effective than monetary incentives with peer referral (SD-M-PR) on average in terms of alters tested and newly-tested alters, despite SD-M-PR having larger effects. (Compared to the control group, the cost for one more alter tester in the SD-M group was $14.90 and $16.61 in the SD-M-PR group. For newly-tested alters, the cost of one more alter in the SD-M group was $24.65 and was $49.07 in the SD-M-PR group.) No study-related adverse events were reported during the study duration. Limitation includes digital network approach might neglect individuals who lack internet access. This trial was registered (ChiCTR1900025433). Analyses were performed on an intention to treat basis. The study design and results were reported according to CONSORT 2010 guidelines.”

**Comment 5:** “At this stage, we ask that you include a short, non-technical Author Summary of your research to make findings accessible to a wide audience that includes both scientists and non-scientists. The Author Summary should immediately follow the Abstract in your revised manuscript. This text is subject to editorial change and should be distinct from the scientific abstract. Please see our author guidelines for more information: https://journals.plos.org/plosmedicine/s/revising-your-manuscript#loc-author-summary”

**Response:** The author summary was added following the abstract, according to your author guidelines:

Author summary

Why Was This Study Done?

	MSM in China have a high burden of HIV while testing coverage remains low.

	HIVST is a practical approach to supplement HIV testing services and reach marginalized individuals who have avoided facility-based testing.

	The strategy of secondary distribution has been adopted by some HIVST programs and proved it can improve HIV testing coverage.

	We aimed to further innovate and promote the current secondary distribution model among MSM in China by adding monetary incentives and peer referral interventions.

What Did the Researchers Do and Find?

	We recruited 309 MSM in a three-arm randomized controlled trial to evaluate the effect of two interventions in promoting the secondary distribution of HIVST between October 2019 to September 2020

	In the two intervention groups, the average number of tested alters motivated by each index was 0.98 in the monetary incentives group and 1.78 in monetary incentives plus peer referral group, compared to 0.57 in the control group.

 The average cost per alter tested in two intervention groups was $61.58 and $41.56, compared to $96.18 in the control group.

What Do These Findings Mean?

	Our digital-based HIVST promotion programs are decentralized and may benefit marginalized populations who avoid facility-based HIV testing due to potential stigma and discrimination.

	Both monetary incentives and monetary incentives plus peer referral can effective engage more individuals to conduct HIVST and increase the HIV testing uptake among MSM.

	The interpretation of our study's findings might not apply to marginalized individuals who have limited access to the internet, and therefore, future studies are needed to address this limitation.

**Comment 6:** “Citations should be in square brackets, and preceding punctuation, throughout the manuscript.”

**Response:** We have edited this throughout the manuscript.

**Comment 7:** “Please update the first line of the legend for Table 1 to ‘Age data are presented as mean (SD). All other data are presented as n (%)'.”

**Response:** Thanks for the comment. We have updated it in the manuscript as per request.

“Age data are presented as mean (SD). All other data are presented as n (%).”

**Comment 8:** “The final figure provided (page 45 – diagram demonstrating principles of standard secondary distribution) does not appear to have a title and is not referenced in the main text.”

**Response:** Sorry for not citing the final figure in the context of the previous submission. The figure title ‘Figure 3. An Infographic of principles of three secondary distribution approaches was added, and the figure was cited in the main text (Line 147).

**Comment 9:** “There are some discrepancies between your original trial registration (https://www.chictr.org.cn/com/25/historyversionpuben.aspx?regno=ChiCTR1900025433) and the manuscript, in terms of inclusion criteria and blinding). Can you please provide the reasoning behind these discrepancies?”

**Response:** Thanks for the comment. In terms of inclusion criteria, there was an error in translation between Chinese and English on the clinical trial registration. We defined one of the inclusion criteria as “willing to order HIVST kits online" in Chinese but mistakenly translated it as “applied to self-tests through local partnering CBO” in English. We have sent a revision request to the trial registration center. In terms of discrepancies of blinding, please kindly find the reasoning as below:

| Item | Trial registration | Manuscript | Reasoning |
| --- | --- | --- | --- |
| Blinding | “The participants will be blinded.” | “This study was only blinded to the statisticians who analyzed the data.” | The reason why participants were unblinded in a real-life setting was because of the intervention of monetary incentives. All group information was listed in the recruitment advertisement and informed consent, and therefore only the statistician was blinded. |

**Comment 10:** “In the discussion, please consider discussing the concept of patients acting as providers - or extending the boundaries of the "health system" to include communities - an innovative and important approach to extending the reach and leverage of social networks, especially to stigmatized populations.”

**Response:** Thanks for the suggestion. We added a discussion point on the concept of patients acting as providers: “Index MSM in this process are not only healthcare seekers but also health providers who may potentially introduce HIV-related knowledge or offer HIVST kits to members within their social networks. The decentralized nature of the secondary distribution of HIVST enables index MSM to get tested and distribute kits outside clinical settings. Therefore, they contribute to cross the boundaries between institutions and community, which is an innovative and effective approach to extend the leverage of social networks and engage marginalized and stigmatized populations [33].”

**Comment 11:** “Please remove the ‘Financial Support’ section from your main text but include this information in your ‘Financial Disclosure’ statement in the submission form. In the event of publication, your form responses will be published as metadata. Please also remove the ‘Declaration of Interests’, ‘Data sharing’, and ‘Author Contributions’ sections from the manuscript main text and ensure that all the information is captured in your responses to the submission form.”

**Response:** Thanks for the comment. We have revised it as per request.

**Comment 12:** “Please correct ‘Reference’ to ‘References’ on page 21.”

**Response:** Thanks for the correction. “Reference” was corrected to “References”.

**Comment 13:** “Please include the analysis plan, with any amendments, in the Supporting Information to be published with the manuscript if accepted.”

**Response:** We have included analysis amendments in protocol amendments in the Supporting Information.

**Comment 14:** “Thank you for completing a CONSORT checklist. Please update the checklist to use section and paragraph numbers, rather than page numbers (as these may change during the revision process).”

**Response:** Thanks for the comment. CONSORT checklist was updated.

**Comment 15:** “Please report your economic analysis according to the CHEERS statement and provide the relevant completed checklist - <https://www.equator-network.org/reporting-guidelines/cheers/>”

**Response:** Thanks for the suggestion. We have reported economic analysis according to the CHEERS statement and attached the checklist in the supporting information.

**Part 2, Response to the comment of Reviewer 1**

**Comment 1:** “Preferred terminology is "assigned male at birth" instead of "born biologically male." Do you know what proportion of the study population identified as cis-men versus trans-women or gender non-conforming?”

**Response:** Thanks for the comment. We have rephrased the language as per suggestion. We did not capture data regarding the gender identity of our study population, and we will include this question in our future research. Rephrased contexts are also listed below.

Abstract: “Between October 21, 2019, and September 14, 2020, a three-arm randomized controlled, single-blinded (statistician) trial was conducted online among 309 individuals (defined as index) who were assigned male at birth, aged 18 years or older, ever had male-to-male sex, willing to order HIVST kits online, and consented to take surveys online.”

Study design: “We planned to recruit 300 eligible participants (100 for each arm) with the following inclusion criteria: 1) assigned male at birth, 2) aged 18 years or older, 3) ever had male-to-male sex, 4) be willing to order HIVST kits online, and 5) be consented to take baseline and follow-up surveys online.”

**Comment 2:** “The authors use a deposit in all arms of their trial, which is a separate mechanism of a financial incentive (contract) and has its mechanistic influence on behavior. Why was this chosen? This should be added to the introduction and discussion section, along with a discussion of how this might impact trial results.”

**Response:** Thanks for the suggestion. We explained why we chose both the incentives and deposit contract in the revised manuscript as follow:

Discussion: In this study, a deposit was required for index individuals to acquire testing kits. The results may differ if the program were implemented without this requirement. Specifically, the effect of monetary incentives could be either larger or smaller depending on whether monetary incentives substitute or complement any motivational effects of the deposit. The marginal effect of incentives could be larger, for instance, if requiring a deposit is already a strong source of motivation (thus leaving less room for financial incentives to have an effect). Deposits could be an independent source of motivation if they acted as a commitment, whereby, after making a deposit, indexes would be motivated by so-called “sunk cost” effects [33]. The effects of a monetary incentive could be smaller without the deposit, however, if the motivational effects of monetary incentives and deposit reinforce one another. Besides, deposits may potentially hinder the participation willingness among those who are aware of loss aversion (89 MSM (21%, 89/417)) consented but unpaid deposit with unknown reasons), it did ensure the overall performance of HIVST and secondary distribution, for instance, it may facilitate the results-returning from testers, which can further benefit the linkage to care and treatments. Deposits may also prompt index participants to order an appropriate number of HIVST kits so that reducing the waste of HIVST kits or over-testing.

**Comment 3:** “The authors note that the number of alters per index might be artificially lowered because of the maximum of 5, 5, and 10 alter slots in each arm. Add details on what percentage of indexes in each arm maxed out the total number of alters allocated to further describe and quantify this potentially artificial ceiling.”

**Response:** Thanks for the comment. The upper limit of the number of kits order might not be a limitation of alters’ motivation. We have provided a supplementary table with details below and have added this information accordingly in the manuscript.

Results: “More information regarding the number of kits ordered and actual kits distribution by index in each group is listed in Supplementary Table 1.”

Supplementary table 1. Summary of total number of ordered kits and actual kits distribution by index participants in each arm (N=309)

|  | **Control***  **N=102 (%)** | **SD-M†**  **N=103 (%)** | **SD-M-PR‡**  **N=104 (%)** |
| --- | --- | --- | --- |
| Number of ordered kits | |  |  |
| 1 | 40 (39) | 29 (28) | 35 (34) |
| 2 | 30 (29) | 29 (28) | 30 (29) |
| 3 | 17 (17) | 17 (17) | 12 (12) |
| 4 | 4 (4) | 3 (3) | 4 (4) |
| 5 | 11 (11) | 25 (24) | 23 (22) |
| Number of actual kits distribution | |  |  |
| 0 | 62 (61) | 54 (52) | 53 (51) |
| 1 | 31 (30) | 27 (26) | 22 (21) |
| 2 | 5 (5) | 4 (4) | 15 (14) |
| 3 | 1 (1) | 8 (8) | 2 (2) |
| 4 | 1 (1) | 8 (8) | 8 (8) |
| 5 | 2 (2) | 2 (2) | 4 (4) |
| >5 | NA | NA | 3 (3) |

*Control refers to standard secondary distribution group. SD-M refers to secondary distribution with monetary incentives group. ‡SD-M-PR refers to secondary distribution with monetary incentives plus peer referral group

**Comment 4:** “I am not familiar with the use of bootstrapping for statistical tests where data are not normally distributed (rather than using a non-parametric test or utilizing regression techniques that have more relaxed assumptions about normality). This should be seen by a statistical reviewer if not addressed in the initial review.”

**Response:** We appreciate this comment. We consulted with our statistician, the co-author (Dr. Zou), and changed statistical tests to zero-inflated negative binomial regression since outcome variables are all counts. This was also suggested by reviewer #4, who is a statistician.

**Comment 5:** “Costing analysis: This is very important, and I commend the authors for including it alongside the preliminary trial results. Table 4 has all of the relevant details for an incremental cost per X analysis, and yet all that is presented in the text is the cost per person tested or cost per person diagnosed, which is not the preferred metric for comparing models. The incremental cost per additional person tested is preferable and present in Table 4 but not discussed.”

**Response:** Thank you for this comment. We have now revised this paragraph to focus on the incremental cost-effectiveness measures. This paragraph now reads as follows:

“Table 4 summarizes the results of the economic evaluation. The total costs were $19,485·97 for 794 testers, including 450 index participants and 344 alter testers. Overall, the average cost per tester was $24·54, and the average cost per alter tester was $56·65. Supplementary Table 3 and Supplementary Figures 1 and 2 give further details of all three groups' fixed and variable costs. Monetary incentives alone (SD-M) were more cost-effective than monetary incentives with peer referral (SD-M-PR) on average in terms of alters tested and newly-tested alters, despite SD-M-PR having larger effects. Compared to the control group, the cost for one more alter tester in the SD-M group was $14.90 and $16.61 in the SD-M-PR group. The incremental cost per additional alter tested in the SD-M-PR group compared to the SD-M group was $17.49. For newly-tested alters, the cost of one more alter in the SD-M group was $24.65 and was $49.07 in the SD-M-PR group. Moving from SD-M to SD-M-R, the incremental cost per newly tested alter was $86.42.”

**Comment 6:** “Recruitment drop off: Any information on the drop off from 415 to 309? What do we know about the 106 who did not consent? What data are there to support that the deposit might be a barrier? This is a substantial fraction of the otherwise eligible population and would need to be described further.”

**Response:** Thanks for the suggestion. We have provided a further detailed explanation in the result and discussion sections. The trial flowchart has been updated accordingly. We have also revised the results part as below.

Results: “In summary, 417 MSM were assessed for eligibility, of whom 1 (0%, 1/417) did not sign the inform consent, 89 (21%, 89/417) were consented but did not pay the deposit with unknown reasons, and 18 (4%, 18/417) were excluded with reasons due to unexpected technical issues or study inclusion criteria. A total of 309 MSM (74%, 309/417) from 68 cities in China were recruited and randomized into control group (n=102), SD-M group (n=103), and SD-M-PR group (n=104), respectively (Figure 1).”

Discussion: ‘In terms of deposit contract, although it may potentially hinder the participation willingness among those who may be loss averse (89 MSM (21%, 89/417) consented but unpaid deposit with unknown reasons), it did ensure the overall performance of HIVST and secondary distribution. For instance, it may facilitate the likelihood to return results from testers, which can further benefit linkage to care and treatment where needed. Deposits may also prompt index participants to order an appropriate number of HIVST kits to reduce waste of HIVST kits or over-testing.’

**Comment 7**: “Line 247 how did the authors get 65 results from 58 unique alters? And same for line 250. This is unclear.”

**Response:** Thank you for your comment. We have explained it in the manuscript to make it clear. The reasoning is as below.

“The discrepancies between the number of used kits and the number of identified alters (control: 65 vs 58; SD-M: 107 vs 101; SD-M-PR: 187 vs 185) are because of alters who tested and uploaded results more than once.”

**Comment 8:** “Line 304 "average cost per tester was $24.54." Is this for index clients or all clients collapsed together? Which costs were the largest drivers? Which were the estimates most sensitive to?”

**Response:** As indicated in the previous sentence, testers include both index participants and alter testers, so $24.54 is for all clients together. We’ve added the cost breakdown for each of the three groups (supplementary figure 2). It shows that fixed cost for staff was the largest cost driver for the control and SD-M groups and that for SD-M-PR group, the highest cost was related to consumables.

“Compared to the control group, the cost for one more alter tester in the SD-M group was $14.90, and that for one extra newly tested alter was $24.65. The average cost per newly tested alter was $348·82 in the control group, $148·08 (58% reduction) in the SD-M group, and $130·31 (63% reduction) in the SD-M-PR group. Similarly, it cost the SD-M-PR group 17.49 for one more alter tester and 86.42 for one newly tested alter tester, compared with the SD-M group. And both SD-M group and SD-M-PR group were dominated by the control group as they incurred higher costs yet produced fewer HIV-reactive results.”

**S2 Figure. Cost breakdown of the three arms of the trial (USD, 2020)**

|  | Fixed-Capital | Fixed-Staff | Fixed-Consumables | Variable-Staff | Variable-Consumables |
| --- | --- | --- | --- | --- | --- |
| Control* | 6129.33 | 17322.3 | 2174.31 | 1382.73 | 10180.9 |
| SD-M† | 6129.33 | 17322.3 | 2174.31 | 1764.06 | 14071.5 |
| SD-M-PR‡ | 6129.33 | 17322.3 | 2174.31 | 2165.34 | 23464.1 |

*Control refers to standard secondary distribution group. †SD-M refers to secondary distribution with monetary incentives group. ‡SD-M-PR refers to secondary distribution with monetary incentives plus peer referral group

**Comment 9:** “Discussion: all of the trial arms include a peer network approach, but what distinguishes third arm is the additional VIRTUAL/ONLINE peer referral instead of in person. This is not well described or clarified in this paper. This should be revised throughout to clarify that peer referral was present in all arms, but the third arm had a virtual referral as well.”

**Response:** Thanks for the suggestion. We added clarification on this in the “Study design and participants” of the method part.

“The trial procedures were similar for the three groups, all of which included a secondary distribution process. What distinguished the control group and intervention groups as a monetary incentive intervention was implemented in the SD-M group and the SD-M-PR group, monetary incentives along with online peer referral, which was a virtual secondary distribution were added as intervention.”

**Comment 10:** “Lines 343-347: how did this speculation compare to observation from the data?”

**Response:** Thanks for the suggestion. Since the statistical methods and results have been updated. We revised this speculation based on the results: “However, our study results also suggested that online peer referral worked in conjunction with monetary incentives. Online peer referral alone had the suboptimal effect of motivating alters. We speculated that monetary incentives were the key contributor to secondary distribution. Nevertheless, due to the incentivized secondary distribution of HIVST, which might potentially cause privacy concerns and limit the flexibility of alters, the strategy of online peer referral further decentralized power to alters of index MSM and allowed them more freedom to determine when, where and who to test with. Simultaneously, index MSM also had more options, both in-person or online peer referral, for distributing HIVST services when their social contacts were reluctant to accept an HIVST test kit due to confidentiality or other concerns caused by in-person contact.

**Comment 11:** “Table 3 is fascinating and should be interpreted further in the results and discussion. Effects more pronounced in older, non-disclosed, and higher risk men.”

**Response:** Thanks for the suggestion. We have updated the results and discussion section of the manuscript.

**Results:** “Higher number of motivated unique alters was associated with index participants who assigned in intervention groups, aged over 30, self-identified as gay, disclosed sexual orientation, previously tested for HIV, and had condomless sex within the past three months. ”

**Discussion:** “Moreover, our subgroup analysis also suggested that interventions impacted differently on MSM with disparate characteristics, including age, sexual orientation, sexual orientation disclosure, HIV testing history, and condomless sex within three months. It provided critical implications on future implementation and optimization of the interventions.”

**Comment 12:** “Figure 2 error bars needed.”

**Response:** Figure 2 has been updated as per request.

### Part 3, Response to the comment of Reviewer 2

**Major comments:**

**Comment 1:** “Deposit refunds were only given upon upload of test results. This could have forced index participants to over test so they could get reimbursed - there were 150 tests from 102 control participants, showing unnecessary testing and potential wastefulness of the intervention. Related to this, in lines 384-389, it does not seem convincing that the restriction on number of kits ordered was actually a limitation as it seems people ordered more than they could distribute”

**Response:** Thanks for the comment. We added further discussion on the deposits in both the introduction and discussion and revised the limitation regarding the restriction. We believe that the combination of monetary incentives and deposit contracts can prompt participants to make an appropriate decision on the number of HIVST they order. In terms of unnecessary testing, index participants are not only the health promoters but also healthcare seekers, as they might also need HIVST themselves. We have added the following content.

Discussion: In this study, a deposit was required for index individuals to acquire testing kits. The results may differ if the program were implemented without this requirement. Specifically, the effect of monetary incentives could be either larger or smaller depending on whether monetary incentives substitute or complement any motivational effects of the deposit. The marginal effect of incentives could be larger, for instance, if requiring a deposit is already a strong source of motivation (thus leaving less room for financial incentives to have an effect). Deposits could be an independent source of motivation if they acted as a commitment, whereby, after making a deposit, indexes would be motivated by so-called “sunk cost” effects [33]. The effects of a monetary incentive could be smaller without the deposit, however, if the motivational effects of monetary incentives and deposit reinforce one another. Besides, deposits may potentially hinder the participation willingness among those who may be loss averse (89 MSM (21%, 89/417)) consented but unpaid deposit with unknown reasons), it did ensure the overall performance of HIVST and secondary distribution. For instance, it may facilitate the likelihood to return results from testers, which can further benefit linkage to care and treatments where needed. Deposits may also prompt index participants to order an appropriate number of HIVST kits to reduce waste of HIVST kits or over-testing.

**Comment 2:** “In the peer referral arm an index could distribute to/refer more people (ten) compared to five in the other arms. This makes results on mean numbers of testers incomparable with the other arms”

**Response:** Thanks for the comment. We understand the reviewer’s concern. This issue can be addressed by our study design. The only difference between monetary incentive and peer referral arm is that the peer referral arm is allowed to do peer referral. And the comparison between these two groups can test the effectiveness of add-in services for HIV prevention. In addition, only 3 out of 104 participants in the SD-M-PR delivered more than five kits, which makes the two groups comparable.

**Comment 3**: “There were baseline differences by arm in participant characteristics. Please comment on potential for confounding”

**Response:** Thanks for the comments. We have provided further details in the manuscript. Study participants were randomized into three groups with an allocation ratio of 1:1:1. There were no baseline differences among the three groups measured by Chi-square tests with p values > 0.05. Please kindly find the information down below.

Results: “Index participants shared similar baseline characteristics with no differences among the three groups (P > 0·05).”

**Table 1· Baseline characteristics of the index participants in Zhuhai, China, 2019-2020 (N= 309)**

|  | Control*  n=102 | SD-M†  n=103 | SD-M-PR‡  n=104 | Overall | P value |
| --- | --- | --- | --- | --- | --- |
| Age (years) | 29·76 (6·02) | 29·83 (7·21) | 29·75 (6·48) | 29.75 (6·57) |  |
| *≤ 30* | 66 (65%) | 67 (65%) | 69 (66%) | 202 (65%) | 0.97 |
| *＞30* | 36 (35%) | 36 (35%) | 35 (34%) | 107 (35%) |  |
| Education |  |  |  |  |  |
| *High school or below* | 20 (20%) | 21 (20%) | 21 (20%) | 62 (20%) | 0.65 |
| *College* | 70 (69%) | 76 (74%) | 75 (72%) | 221 (72%) |  |
| *Master’s degree or above* | 12 (12%) | 6 (6%) | 8 (8%) | 26 (8%) |  |
| Monthly Income (USD, $) |  |  |  |  |  |
| *<225* | 8 (8%) | 8 (8%) | 8 (8%) | 24 (8%) | 0.24 |
| *225-450* | 7 (7%) | 7 (7%) | 5 (5%) | 19 (6%) |  |
| *451-750* | 16 (16%) | 34 (33%) | 24 (23%) | 74 (24%) |  |
| *751-1200* | 37 (36%) | 27 (26%) | 39 (38%) | 103 (33%) |  |
| *>1200* | 34 (33%) | 27 (26%) | 28 (27%) | 89 (29%) |  |
| Sexual Orientation |  |  |  |  |  |
| *Gay* | 73 (72%) | 68 (66%) | 80 (77%) | 221 (72%) | 0.22 |
| *Others§* | 29 (28%) | 35 (34%) | 24 (23%) | 88 (28%) |  |
| Sexual Orientation Disclosure\| |  |  |  |  |  |
| *Yes* | 57 (56%) | 58 (56%) | 55 (53%) | 170 (55%) | 0.86 |
| *No* | 45 (44%) | 45 (44%) | 49 (47%) | 139 (45%) |  |
| Marital Status |  |  |  |  |  |
| *Single* | 87 (85%) | 87 (85%) | 87 (84%) | 261 (84%) | 0.39 |
| *Engaged or married* | 14 (14%) | 10 (10%) | 13 (13%) | 37 (12%) |  |
| *Separated or divorced* | 1 (1%) | 6 (6%) | 4 (4%) | 11 (4%) |  |
| Residence |  |  |  |  |  |
| *Guangdong province* | 73 (72%) | 71 (69%) | 67 (64%) | 211 (68%) | 0.54 |
| *Other provinces* | 29 (28%) | 32 (31%) | 37 (36%) | 98 (32%) |  |
| Condomless sex in the past 3 months# |  |  |  |  |  |
| *Yes* | 35 (34%) | 39 (38%) | 33 (32%) | 107 (35%) | 0.65 |
| *No* | 67 (66%) | 64 (62%) | 71 (68%) | 202 (65%) |  |
| Ever tested for HIV |  |  |  |  |  |
| *Yes* | 87 (85%) | 89 (86%) | 86 (83%) | 262 (85%) | 0.75 |
| *No* | 15 (15%) | 14 (14%) | 18 (17%) | 47 (15%) |  |

Age data are presented as mean (SD). All other data are presented as n (%). *Control refers to standard secondary distribution group. †SD-M refers to secondary distribution with monetary incentives group. ‡SD-M-PR refers to secondary distribution with monetary incentives plus peer referral group. §Others include heterosexual, bisexual, and not sure. |Sexual Orientation Disclosure refers to whether an index disclose his sexual orientation to people other than sexual partners, e·g· healthcare providers, friends, family members etc. #Condomless sex in the past months refers to whether an index had condomless sex with another man in the past three months.

**Minor comments**
**Comment 4:** “Consider the rewording the definition of alters because in the abstract it sounds like an alter is the whole network/group, rather than one individual”

**Response:** Thanks for the comment. We have revised the language in the abstract and provided it below.

“Members who received kits through the index MSM or the peer referral links from the index MSM were defined as alters.”

**Comment 5:** “Although it is clear in the manuscript, in the abstract it is unclear where self-test results were uploaded (line 16)”

**Response:** Thanks for the comment. We have added details in the abstract as per suggestion and copied the updated version down below.

“Index participants in the two intervention groups could receive a fixed incentive ($3 USD) for the verified test result uploaded back to the digital platform by each unique alter.”

**Comment 6:** “From the abstract it is not immediately clear what is being referred to as the digital approach”

**Response:** Thanks for the comment. We have added further details of the digital approach in the abstract, including completing surveys, ordering HIVST kits, ordering peer-referral links, uploading test results, and receiving incentives.

Abstract: “Between October 21, 2019, and September 14, 2020, a three-arm randomized controlled, single-blinded (statistician) trial was conducted online among 309 individuals (defined as index) who were assigned male at birth, aged 18 years or older, ever had male-to-male sex, willing to order HIVST kits online, and consented to take surveys online. Index participants were randomly assigned into one of the three arms: 1) standard secondary distribution (control) group (n=102), 2) secondary distribution with monetary incentives (SD-M) group (n=103), and 3) secondary distribution with monetary incentives plus peer referral (SD-M-PR) group (n=104). Index participants in the three groups were encouraged to order HIVST kits online and distribute them to members within their social networks. Members who received kits were defined as alters. Index participants in the two intervention groups could receive a fixed incentive ($3 USD) online for the verified test result uploaded back to the digital platform by each unique alter. Index participants in the SD-M-PR group could also share a personalized peer referral link with alters to order kits online. All index participants were assigned an online 3-month follow-up survey after ordering their kits.”

**Comment 7**: “Page 13 lines 81-82: it is not clear what "may crowd them out" means”

**Response:** Thanks for the comment. We removed the jargon and explained it more easily:

‘The effectiveness of secondary distribution programs, on the other hand, may depend more on intrinsic pro-social preferences (or altruistic motives) of those distributing kits to peers [18], and monetary incentives could backfire. Thus, extrinsic monetary incentives could either enhance or diminish intrinsic motivation to distribute kits [19,20].’

**Comment 8:** “Line 158 - not clear whether the index received an additional 20rmb to the one described in line 157”

**Response:** An index can receive an additional 20 RMB for a successful referral. We revised these lines to ‘Index participants could receive monetary incentives for self-tests and distribution. They could receive 20 RMB (≈$3) for self-tests. In terms of distribution, both the index participant and the alter could receive 20 RMB when self-test results were uploaded and verified by each unique alter (by checking the confirmation code and phone number).

**Comment 9:** “Line 182 - Syphilis comes from nowhere without having been described in the methods nor any of the earlier sections. How and where were syphilis tests done?”

**Response:** Thanks for the comment. Syphilis was tested concurrently with HIV as we participants were provided with the dual test kit. We revised the study procedures in line 173-174: ‘In this group, index participants completed the baseline survey and ordered HIV/Syphilis dual rapid test kits (SD Bioline Company, South Korea) through the online system.’

**Part 4, Response to the comment of Reviewer 3**

**Comment 1:** “Rationale for the study: The study is based on the fact that only about half of MSM in China are aware of their HIV status despite a known high prevalence (>5%) in previous studies. The second justification is that COVID has limited face to face services that would routinely be used to improve the yield of testing services leaving few options available. While HIV ST approaches have been successfully in increasing the reach of testing and linkage to care including ART initiation most studies are based in Africa. The author should indicate that there is a reasonable expectation that this will be the case in the context of China which has very different sociocultural context and levels of HIV associated stigma.”

**Response:** Thanks for the suggestion. A rationale regarding HIV associated stigma was added in the introduction as follow:

‘HIV self-testing (HIVST) has been shown as an effective approach to supplement the HIV testing services and reach individuals who have avoided facility-based testing [5]. Both during the COVID-19 pandemic and in the long run, HIVST can address unique programmatic needs and global HIV testing targets [6]. Particularly, in China, studies suggested that HIV-phobia and institutional discrimination against people living with HIV are common [7]. The confidential and private nature of HIVST has potential for addressing stigma and discrimination. More importantly, HIVST as an innovative method can be expected to not only reach but also link people to care [8].’

**Comment 2:** “Sample size: This is provided based on findings from a pilot study. The standard assumptions of power (> 80%), alpha (0.05) for the minimum detectable effect size are clearly provided. Randomization: This was done by computer generated randomization. The author should comment on whether block sizes with variable block sizes or otherwise was employed.”

**Response:** Thanks for the comment. We have added further details of randomization in the manuscript.

Randomization and masking: “Eligible participants were randomly assigned to one of the three arms individually and independently by a computer-generated program which based on a 1:1:1 allocation ratio.”

**Comment 3:** “Allocation concealment: This is not described and should be included.”

**Response:** Thanks for the comment. We have added further details in the manuscript.

“Allocation concealment was ensured, as randomization took place before the interventions, and participants could not switch study arms later on.”

**Comment 4:** “Harms: There is no deliberate reporting of harms. Specifically the authors should indicate whether or not there were any cases of social harms related to HIV testing and diagnosis.”

**Response:** Thanks for the suggestion. We have added details of the harms in the manuscript.

“No study-related adverse event was reported within the trial duration.”

**Comment 5:** “Discussion: This reflects the importance of the study findings. An additional limitation is generalizability beyond the period of COVID with its mitigation measures. For instance it is unclear to what extend the same monetary incentive will hold after economic recovery.”

**Response:** We understand the concern from the reviewer and have listed this as a limitation of the study. However, we must clarify that the differences between COVID-19 and post COVID-19 would be small, as the vast majority of MSM testers already moved to self-testing (75% of men who tested in the last six months used self-testing), and this trend continues to increase in China. We believe that the high self-testing rate will be sustained, even after economic recovery, which is evidenced by our series of annual surveys.

**Part 5, Response to the comment of Reviewer 4**

**Comment 1:** “The main issue is that the dependent variables are counts. Therefore, count regression models should be used. The authors could try Poisson regression but will likely want to move to negative binomial regression. as the assumptions of Poisson are rarely met. Simply taking confidence intervals and comparing them isn't adequate. A regression model would also allow the use of control variables such as age.  The economic analysis should probably use OLS regression or maybe quantile regression.”

**Response:** We agree with the reviewer. We further compared Poisson regression and negative binomial regress. We found that negative binomial regression is more suitable as the variance of the count number is larger than the mean value, and the assumption of Poisson regression are not met. More specifically, we have tried Zero-inflated negative binomial regression as the count number data exhibits some dispersion and excess zeros. As for OLS regression, there’s no regression involved with the comparative cost-effectiveness other than taking the estimated treatment effects from the treatment regressions. We have updated outcome analyses in the manuscript and are also provided below.

**Comment 2:** “p. 3 lines 28-30  (and also in the main text): Instead of giving mean and sd, please give median and IQR. Clearly, these variables are highly skewed and negative values are impossible. In addition, tables of the number of alters by group should be given (something like:

                         Control          SDM       SDMPR
0
1
2
3

etc.”

**Response:** Thanks for the comment. We have updated IQR in table 2, provided a summary table with details below, and added this information accordingly in the manuscript.

Supplementary table. Summary of the total number of ordered kits and actual kits distribution by index participants in each arm (N=309)

|  | **Control***  **N=102 (%)** | **SD-M†**  **N=103 (%)** | **SD-M-PR‡**  **N=104 (%)** |
| --- | --- | --- | --- |
| Number of ordered kits | |  |  |
| 1 | 40 (39) | 29 (28) | 35 (34) |
| 2 | 30 (29) | 29 (28) | 30 (29) |
| 3 | 17 (17) | 17 (17) | 12 (12) |
| 4 | 4 (4) | 3 (3) | 4 (4) |
| 5 | 11 (11) | 25 (24) | 23 (22) |
| Number of actual kits distribution | |  |  |
| 0 | 62 (61) | 54 (52) | 53 (51) |
| 1 | 31 (30) | 27 (26) | 22 (21) |
| 2 | 5 (5) | 4 (4) | 15 (14) |
| 3 | 1 (1) | 8 (8) | 2 (2) |
| 4 | 1 (1) | 8 (8) | 8 (8) |
| 5 | 2 (2) | 2 (2) | 4 (4) |
| >5 | NA | NA | 3 (3) |

*Control refers to standard secondary distribution group. SD-M refers to secondary distribution with monetary incentives group. ‡SD-M-PR refers to secondary distribution with monetary incentives plus peer referral group

**Comment 3:** “Starting on p 9 line 177:  Remove each use of "mean".  The outcome variable is the number of alters, not the mean number of alters. A variable should exist for each observation; here, each index person has a number of alters.”

**Response:** Thanks for the comment. We have removed each use of “mean” in the manuscript.
