# Supplementary material for: Monetary incentives and peer referral in promoting secondary distribution of HIV self-testing among men who have sex with men in China: A randomized controlled trial
Source: PLoS Med. 2022 Feb 14;19(2):e1003928. doi: 10.1371/journal.pmed.1003928 (PMC8887971; doi:10.1371/journal.pmed.1003928)
Supplement: Attachment — Submitted filename: Response letter_23Nov21_.docx [file pmed.1003928.s016.docx]

Dear Dr. Davidson,

Thank you again for the invaluable comments. We have revised the manuscript according to the comments. Please find our point-to-point responses, a marked-up copy of the revised manuscript, and a clean copy of the revised manuscript.

Weiming Tang, Ying Lu and Yuxin Ni on behalf of the team

——————————————————————————————
**Part 1, Response to the comments from the Editors**

**Comment 1:** “Please provide details of who funded the study in your Financial Disclosure (the information removed from the previous version ought to be captured as part of your response to the submission form rather than removed entirely).”

**Response**: Thanks for the guidance. We have provided details accordingly in the submission form.

Financial Disclosure: This work was supported by the National Nature Science Foundation of China [grant numbers 81903371], the National Key Research and Development Program of China [grant number 2017YFE0103800], Academy of Medical Sciences and the Newton Fund [grant number NIF\R1\181020], the National Institutes of Health [R34MH119963, R25 AI140495]; UNC Center for AIDS Research [grant number NIAID 5P30AI050410], and Zhuhai Medical and Health Science and Technology Plan Project [grant number 20181117A010064].

**Comment 2**: “Please define the abbreviations MSM and HIVST in your Author Summary.”

**Response:** Thanks for the suggestion. We have defined MSM and HIVST in the Author Summary.

- Men who have sex with men (MSM**)** in China have a high burden of HIV while testing coverage remains low.
- HIV self-testing **(**HIVST**)** is an effective approach to supplement HIV testing services and engage marginalized individuals who have avoided facility-based testing due to potential stigma and discrimination.

**Comment 3:** “Please ensure your Author Summary can be understood without the need to consult the main text (e.g. briefly define your intervention groups and avoid jargon where possible).”

**Response:** Thanks for the comment. We have explained the intervention groups and briefly defined “secondary distribution” and “digital-based”.

“Author Summary

Why Was This Study Done?

	Men who have sex with men (MSM) in China have a high burden of HIV while testing coverage remains low.

	HIV self-testing (HIVST) is an effective approach to supplement HIV testing services and engage marginalized individuals who have avoided facility-based testing due to potential stigma and discrimination.

	The strategy of secondary distribution, whereby individuals receive multiple HIVST kits and distribute them to members within their social networks, including sexual partners and friends, has been adopted by some HIVST programs and proved it can improve HIV testing coverage.

	We aimed to further innovate and promote the current secondary distribution model to a digital-based one among MSM in China, where they can order HIVST online. We also added interventions of monetary incentives and peer referral to evaluate whether they can amplify the effectiveness of secondary distribution.

What Did the Researchers Do and Find?

	We recruited 309 participants to evaluate the effect of two interventions: 1) monetary incentives group; 2) monetary incentives plus peer referral group in promoting secondary distribution of HIVST compared to the control group, between October 2019, to September 2020

	In the two intervention groups, the number of tested alters motivated by each index was 0.98 in monetary incentives group and 1.78 in monetary incentives plus peer referral group, compared to 0.57 in the control group.

	The average cost per alter tested was $61.58 in monetary incentives group and $41.56 in monetary incentives plus peer referral group, compared to $96.18 in the control group.

What Do These Findings Mean?

	Our digital-based HIVST promotion programs are decentralized and may benefit the wider marginalized populations who avoid facility-based HIV testing.

	Both monetary incentives and monetary incentives plus peer referral can effectively engage more individuals to conduct HIVST and increase the HIV testing uptake among MSM.

	The interpretation of our study's findings might not apply to marginalized individuals who have limited access to the internet, and therefore, future studies are needed to address this limitation.”

**Comment 4:** “In the Author Summary, under 'What Do These Findings Mean' bullet point 2, please correct 'effective' to 'effectively'.”

**Response:** Thanks for the comment. We have corrected it to ‘effectively’.

“Both monetary incentives and monetary incentives plus peer referral can effectively engage more individuals to conduct HIVST and increase the HIV testing uptake among MSM”.

**Comment 5:** “Please cite both your CONSORT and CHEERS checklists in your methods so readers know they can be found in the supplementary materials (currently reads 'available on request').”

**Response:** Thanks for the suggestion. We have cited both checklists in the statistical analysis section of methods.

“This study was registered in the Chinese Clinical Trial Registry (ChiCTR1900025433). Results reporting followed the extension of the CONSORT 2010 statement [29] (S1 File. CONSORT 2010 Checklist). The economic analysis was reported according to the CHEERS statement [30] (S2 File. CHEERS Statement).”

**Comment 6:** “In the flow diagram (Figure 1), please indicate the number of individuals in each group analyzed in the ITT analysis.”

**Response:** Thank you for the suggestion. We have added an additional note in the flow diagram (Figure 1).

“The number of index participants analysed in the intention-to-treat was 102 in the control group, 103 in the SD-M group, and 104 in the SD-M-PR group”.

Comment 7: “Please update your CONSORT and CHEERS checklists to use section name and paragraph number (e.g. Discussion, Paragraph 2) rather than page number - page numbers change frequently during the revision process.”

**Response:** Thanks for the suggestion. We have revised the checklists using section names and paragraph numbers.

**Comment 8:** “All information in your 'Declarations' file (supplementary material) should either be captured as part of your responses to the submission form (Declarations of Interests, Data availability, Author contributions) or should be relocated to the end of the main text (Acknowledgments).”

**Response:** Thanks for the guidance. We have added related information to the submission form as per request.

**Part 2, Response to the comment of Reviewer 1**

**Comment 1:** “The wording is unclear in several sections that have been rewritten, including a) the description of the deposit mechanism in the discussion, b) the description of the virtual/online peer referral in the third arm with peer referral.”

**Response:** Thanks for the suggestion. We further revised the two parts and tried to make them clearer. In the discussion's description of the deposit mechanism, we have reordered some statements to make them more logical and easier to understand. In the description of the peer referral arm in the methods, we have explained more about this intervention.

a): “In this study, a deposit was required for index individuals to acquire testing kits. The results may differ if implemented the program without this requirement. Specifically, the effect of monetary incentives could be either larger or smaller depending on whether monetary incentives substitute or complement any motivational effects of the deposit. Deposits could be an independent source of motivation if they acted as a commitment, whereby, after making a deposit, indexes would be motivated by so-called “sunk cost” effects [34]. The marginal effect of incentives could be more prominent if requiring a deposit is already a strong source of motivation (thus leaving less room for financial incentives to have notable effects). However, the effects of a monetary incentive could be smaller without the deposit if the motivational effects of monetary incentives and deposit reinforce one another. Besides, deposits may potentially hinder the participation willingness among those who may be loss averse (89 MSM (21%, 89/417)) consented but unpaid deposit with unknown reasons), it did ensure the overall performance of HIVST and secondary distribution. For instance, it may facilitate the likelihood to return results from testers, which can further benefit linkage to care and treatments where needed. Deposits may also prompt index participants to order appropriate number of HIVST kits to reduce waste of HIVST kits or over-testing.”

b): “*SD-M-PR group: secondary distribution with monetary incentives plus peer referral*

Participants in this arm were provided with the option of online peer referral, in addition to the monetary incentive arm procedures as previously described. Except for ordering and distributing kits directly to the alters, index participants in this arm could also utilize the approach of peer referral to complete the secondary distribution virtually. Index participants received a unique and personalized peer referral link to share with the alters, and they could distribute up to 5 HIVST kits through this channel (each alter could order one test kit through the link). In the system of peer referral, referred alters clicked the link shared by the index participant and completed the process of ordering and returning all by themselves. Alters paid the refundable deposit of 100 RMB ($15) online, provided the address, received the testing kits, conducted self-tests, and uploaded the results back to the platform. Similarly, both index participants and alters received a 20 RMB ($3) for verified tests by a unique alter. In this group, index participants could refer to up to 10 alters (5 kits distribution + 5 peer referral) and receive a maximum of 200 RMB.”

**Comment 2:** “The authors responded to the question about whether the number of alters per index might be artificially lowered because of the maximum of 5, 5, and 10 alter slots in each arm. They added Supplementary table 1 and a narrative interpretation. However, Supplementary table 1 only shows the 5+ category in the bottom half of the table about the number of test kits distributed, rather than ordered, which prohibits assessing how many ordered >5 kits. Then, the number of kits distributed was higher in the SD-M-PR arm (as per the table), so the interpretation is inaccurate: "the upper limit of the number of kits order[ed] might not be a limitation of alters' motivation". This section should be revised for data completeness and accuracy.”

**Response:** Thank you for your comment. The maximum number of ordered kits was the same (5) in each group. In the SD-M-PR group, index participants could request virtual peer-referral links and share links with alters who could use links to order kits that were not included in the number of ordered kits from index participants, as they do not need to order these by themselves. We have added this information to the notes of supplementary table 1 to make it more clear, and have changed the text to reflect this.

**Comment 3:** “The abstract was fully revised to remove much of the primary results of the paper in favor of a lot of details about the costing analysis; why were the primary results cut?”

**Response:** Thanks for the comment, we removed some of the primary results because of the word limit. We have added primary results back in the abstract without considering the word limit.

“The number of unique tested alters motivated by each index was 0.57±0.96 in the control group, compared with 0.98±1.38 in the SD-M group (mean difference [MD] 0.41, 95% CI: 0.08-0.74), and 1.78±2.05 in the SD-M-PR group (MD=1.21, 95% CI: 0.77-1.65). The number of newly tested alters motivated by each index was 0.16±0.39 in the control group, compared with 0.41±0.73 in the SD-M group (MD=0.25, 95% CI: 0.09-0.41), and 0.57±0.91 in the SD-M-PR group (MD=0.41, 95% CI: 0.22-0.60), respectively.”

**Comment 4:** “The main point about the second arm including peer referral (in person) and the third arm including VIRTUAL or DIGITAL peer referral is still really missing in the paper and erroneously makes it seem as though "peer referral" (instead of "virtual peer referral") is the unique concept being tested in the third arm. This point was made nicely on page 8 in response to my prior comment, but then throughout the rest of the manuscript the same clarity is missing.”

**Response:** Thanks for the comment. We have rewritten the SD-M-PR arm in the methods section to reflect what we have responded before.

“Participants in this arm were provided with the option of online peer referral, in addition to the monetary incentive arm procedures as previously described. Except for ordering and distributing kits directly to the alters, index participants in this arm could also utilize the approach of peer referral to complete the secondary distribution virtually. Index participants received a unique and personalized peer referral link to share with the alters, and they could distribute up to 5 HIVST kits through this channel (each alter could order one test kit through the link). In the system of peer referral, referred alters clicked the link shared by the index participant and completed the process of ordering and returning all by themselves. Alters paid the refundable deposit of 100 RMB ($15) online, provided the address, received the testing kits, conducted self-tests, and uploaded the results back to the platform. Similarly, both index participants and alters received a 20 RMB ($3) for verified tests by a unique alter. In this group, index participants could refer to up to 10 alters (5 kits distribution + 5 peer referral) and receive a maximum of 200 RMB.”

**Comment 5:** “Page 16 of the marked-up manuscript, line 430 says "similarly" to compare two sets of outcomes in which one has an association and the other does not; is there a typo in the interpretation?”

**Response:** Thank you for the comment, we have deleted “Similarly” as it was a typo.

**Comment 6:** “Page 16 of the marked-up manuscript, most of the absolute value changes and differences between arms have been removed, leaving only the relative changes (IRR). The absolute differences are important, even if only means without bootstrapped 95%Cis.”

**Response:** Thank you for the comment, we have added mean difference into the table and revised in the manuscript accordingly.

“In the control group, the number of tested alters motivated by index was 0.57 (SD=0.96), compared with 0.98 (SD=1.38) in SD-M with a mean difference of 0.41, and 1.78 (SD=2.05) in SD-M-PR with a mean difference of 1.21, respectively.”

“The number of newly tested motivated by index was 0.16 (SD=0.39) in the control group, compared with 0.41 (SD=0.73) in SD-M with a mean difference of 0.25, and 0.57 (SD=0.91) in SD-M-PR with a mean difference of 0.41.”

**Table 2. Outcome analysis of 309 index participants in China at the end of the study, 2019-2020**

| **Outcomes** |  | **Number of unique alters motivated by each index participant** | **Number of newly-tested alters motivated by each index participant** | **Number of alters with HIV-reactive results identified by each index participant** |
| --- | --- | --- | --- | --- |
| **Groups, n**  **(mean, SD)** | Control* (N=102) | 58 (0.57, 0.96) | 16 (0.16, 0.39) | 6 (0.06, 0.24) |
|  | SD-M† (N=103) | 101 (0.98, 1.38) | 42 (0.41, 0.73) | 4 (0.04, 0.19) |
|  | SD-M-PR‡ (N=104) | 185 (1.78, 2.05) | 59 (0.57, 0.91) | 5 (0.05, 0.17) |
| **Mean Difference** | Control (ref) vs SD-M | 0.41 | 0.25 | -0.02 |
|  | Control (ref) vs SD-M-PR | 1.21 | 0.41 | -0.01 |
|  | SD-M (ref) vs SD-M-PR | 0.80 | 0.16 | 0.01 |
| **Models, IRR/OR (95% CI), p-value** | Control (ref) vs SD-M | 2.98 (1.82, 4.89) *** | 4.22 (1.93, 9.23) *** | 0.79 (0.18, 3.27) |
|  | Control (ref) vs SD-M-PR | 3.26 (2.29, 4.63) *** | 3.49 (1.92, 6.37) *** | 0.79 (0.21, 2.89) |
|  | SD-M (ref) vs SD-M-PR | 1.25 (0.87, 1.81) | 1.45 (0.58, 3.61) | 1.19 (0.25, 6.24) |

*Control refers to standard secondary distribution group. †SD-M refers to secondary distribution with monetary incentives group. ‡SD-M-PR refers to secondary distribution with monetary incentives plus peer referral group. 0R = odds ratio, IRR = incidence rate ratio. Zero inflated negative binomial regression was used to calculate IRR for outcomes “number of unique alters tested” and “number of newly-tested alters for HIV”. Logistic regression was used to calculate OR for outcome “Number of alters identified with HIV-reactive results”. IRR or OR were all adjusted for age, income, education, marital status, sexual orientation, and residence. **p < 0.01, ***p < 0.001.

**Comment 7**: “The titles for Table 2 and 3 are mixed in the text. Subgroup analyses are Table 3, not 2.”

**Response:** Thanks for the comment and sorry for the confusion. What we want to say is “The group to which an index participant was randomly assigned was not associated with the number of alters with HIV-reactive results identified by each index (Table 2).”, which is not subgroup analyses. We have revised the language in the main text accordingly.

**Comment 8:** “The directionality of the association of disclosure of sexual orientation and tested alters is different depending on the comparison groups; the text should reflect this mixed directionality and does not.”

**Response:** Thank you for pointing this out. We have revised the manuscript accordingly.

“Compared to those in the control group, index participants (SD-M-PR group) who disclosed sexual orientation had a significantly positive association with the number of unique alters, while index participants in the SD-M group had a significant negative association (Table 3).”

**Comment 9:** “I would ask the authors to re-interpret their cost-effectiveness section of the text, especially the wording related to more or less cost-effective. The table is correct but doesn't show the SD-M-PR arm compared to the control arm to support the text interpretation.”

**Response:** Thanks for the comment. We have re-interpreted the cost-effectiveness outcomes in Results and Discussion sections.

Results: “Table 4 summarizes the results of the economic evaluation. The total program costs were estimated to be $19,485.97 for t794 testers, including 450 index participants and 344 alter testers. Overall, the average cost per tester was $24.54, and the average cost per alter tester was $56.65. Supplementary Table 3 and Supplementary Figures 1 and 2 give further details of all three groups' fixed and variable costs. The average cost per tester in the SD-M group was $24.20 compared with $23.44 in the SD-M-PR group, and $26.69 per tester in the control group. For alter testers, the mean costs in the SD-M and SD-M-PR groups were $61.58 and $41.56, respectively, whereas that in the control group was $96.18. In addition, the average costs for newly alter testers increased substantially to more than $100 in all three groups (Table 4). Compared to the control group, the cost for one more alter tester in the SD-M group was $14.90, and $16.61 in the SD-M-PR group. The incremental cost per additional alter tested in the SD-M-PR group compared to the SD-M group was $17.49. For newly-tested alters, the cost of one more alter in the SD-M group was $24.65 and was $49.07 in the SD-M-PR group, compared to the controls. Moving from SD-M to SD-M-R, the incremental cost per newly tested alter was $86.42. Both SD-M group and SD-M-PR group were dominated by the control group as they incurred higher costs yet produced fewer HIV-reactive results.”

Discussion: “Additionally, our economic evaluation suggested that, compared to standard secondary distribution, provision of small monetary incentives to index MSM lead to more alter testers and new alter testers getting tested, therefore lowering the average costs for each alter tested and new alter. Meanwhile, the cost for one additional tester was the smallest in secondary distribution with monetary incentives (SD-M) arm when compared with the standard secondary distribution arm. This implied that this strategy would be cost-effective given a cost-effectiveness threshold of no less than $13.35 per tester. For programs with a budget of more than $7688·30 and a cost-effectiveness threshold of no less than $17.73 per tester, secondary distribution with monetary incentives and peer referral (SD-M-PR) would be the preferred strategy as it could engage the most testers at a relatively low incremental cost. Adding monetary incentives was, however, more expensive than non-incentivized secondary distribution to identify an alter tester with a new HIV diagnosis. However, given the low numbers in the trial, there is uncertainty surrounding for this finding. Our economic data may help inform HIVST secondary distribution interventions in similar contexts. For those focusing on expanding HIVST using secondary distribution method in resource restricting settings, adding a small monetary incentive to motivate index individuals holds promise to further amplify the value of distributing HIVST within their social networks. However, the generalizability of the economic findings in this study should be further examined by sensitivity analysis.”

### Part 3, Response to the comment of Reviewer 2

**Major comments:**

**Comment 1:** “The response to Comment 1 from Reviewer 2 still does not address the difference in numbers of tests among index participants and the number of index participants, and the likely over-testing. It would be important to spell out over what time/period the assessments of testing uptake were made for each index. Surveys were conducted three months after randomisation, were assessments of testing uptake also done over three months, in which case unnecessary testing was possible - there were more index testers than index participants, e.g. there were 150 tests received from 103 SD-M index participants?”

**Response:** Thanks for the comment, we have added the details regarding when to assess testing uptake in the manuscript.

“We assessed testing uptake rates and administered a follow-up survey three months after index participants ordering.”

Regarding the unnecessary testing, we further emphasized this in the limitation part of the study.

“Fourth, unnecessary testing may occur during the experiment because multiple kits might be ordered, and index participants were expected to use and distribute them within three months. However, the original intent of that permitted the individuals to order multiple kits was to encourage index participants to complete secondary distributions. ”

**Comment 2:** “Related to above, in response to comment 7 from reviewer 1, authors state that there were discrepancies related to people who uploaded results more than once. It would be expected that the study methods would be able to correct such duplication.”

**Response:** Thanks for the comment, for testers who uploaded results more than once, we removed duplication based on their phone numbers and used their most recent results for data analysis. We have added the details to the manuscript.

**Comment 3**: “The response to Reviewer 3 on allocation concealment does not seem to address the question. How was allocation concealment done? Who told the participants what arm they were in (and how was this done) and how was the computer-generated code concealed from study staff? Was this all done electronically without need for staff interaction? The methods of recruitment and allocation are unclear”

**Response:** Thanks for the comment, we have added further details in randomization and masking section of the methods.

“Index participants were asked to play a spinning game at the beginning of the baseline survey, which was to assign them into one of the groups randomly based solely on a 1:1:1 allocation ratio. Each participant can only play the game once based on the IP address and phone number. Both study staff and index participants knew about the group assignment at the same time when index participants submitted the baseline survey. Both study staff and index participants were unblinded to allocation, and they knew about the group assignment at the same time when index participants completed the baseline survey. The group allocations remained concealed as the randomization took place before the interventions, and participants could not switch study arms later. Only the statisticians who analyzed the data remained blind to participant allocations.”

**Minor comment**
**Comment 4:** “Page 11 Author summary: Under the paragraph "What do these findings mean", the term 'digital based programs' comes out of nowhere as the preceding paragraphs haven't mentioned that the programs are digital based.”

**Response:** Thanks for the comment, we have explained more about our digital-based program in this section.

“We aimed to further innovate and promote the current secondary distribution model to a digital-based one among MSM in China, where they can order HIVST online. We also added interventions of monetary incentives and peer referral to evaluate whether they can amplify the effectiveness of secondary distribution.”
