# Supplementary material for: Monetary incentives and peer referral in promoting secondary distribution of HIV self-testing among men who have sex with men in China: A randomized controlled trial
Source: PLoS Med. 2022 Feb 14;19(2):e1003928. doi: 10.1371/journal.pmed.1003928 (PMC8887971; doi:10.1371/journal.pmed.1003928)
Supplement: Attachment — Submitted filename: Response letter_22Dec21.docx [file pmed.1003928.s017.docx]

Dear Dr. Davidson,

Thank you again for the invaluable comments. We have revised the manuscript according to the comments. Please find our point-to-point responses, a marked-up copy of the revised manuscript, and a clean copy of the revised manuscript.

Weiming Tang, Ying Lu and Yuxin Ni on behalf of the team

——————————————————————————————
**Part 1, Response to the comments from the Editors**

**Comment 1:** “Please clearly state and define the primary outcomes of the study in the abstract.”

**Response**: Thanks for the suggestion. We have revised the abstract accordingly.

“The primary outcomes were assessed using zero-inflated negative binomial regression to determine the group differences in the number of alters and the number of newly tested alters motivated by each index.”

**Comment 2**: “In the abstract, please include effect sizes and 95% CI for the primary outcomes (those found in the lowermost rows of Table 2).”

**Response:** Thanks for the comment. We have added effect sizes and 95% CI accordingly in the abstract.

**“**Results indicated that index participants in intervention arms were more likely to motivate unique tested alters (SD-M vs. control: IRR = 2.98, 95% CI = 1.82 to 4.89, p-value < 0.001; SD-M-PR vs. control: IRR = 3.26, 95% CI = 2.29 to 4.63, p-value < 0.001) and newly-tested alters (SD-M vs.control : IRR = 4.22, 95% CI = 1.93 to 9.23, p-value < 0.001; SD-M-PR vs.control : IRR = 3.49, 95% CI = 1.92 to 6.37, p-value < 0.001) to conduct HIVST.**”**

**Comment 3:** “Please remove the sentence 'The study design and reporting of the results were followed the CONSORT 2010 guidelines' from the abstract.”

**Response:** Thanks for the suggestion. We have removed the sentence from the abstract.

**Comment 4:** “Please include the numbers of new HIV infections in the abstract.”

**Response:** Thanks for the comment. We have added the information in the abstract.

“Fifteen alters tested with HIV-reactive results.”

**Comment 5:** “Please remove p-values from Table 1.”

**Response:** Thanks for the comment. The column of p-values from Table 1 has been removed.

**Comment 6:** “Please amend 'newly testers' in the title of Figure 2.”

**Response:** Thanks for the comment. We have revised the title to “newly tested alters” accordingly.

**Comment 7:** “Throughout the manuscript, please report relevant p-values after 95% CI.”

**Response:** Thanks for the comment. We have revised the results section of the manuscript accordingly.

“Compared with index participants in the control group, index participants in intervention groups were more likely to motivate more unique alters to self-tested for HIV (control vs. SD-M: IRR = 2.98, 95% CI = 1.82 to 4.89, **p-value < 0.001**; control vs. SD-M-PR: IRR = 3.26, 95% CI = 2.29 to 4.63, **p-value < 0.001**, respectively). The number of unique alters motivated by each index was not associated with the assignment of index participants to the SD-M group or the SD-M-PR group (IRR = 1.25, 95% CI = 0.87 to 1.81, **p-value > 0.05**). …….

The likelihood that the number of newly-tested alters motivated by each index participant was significantly increased when index participants were in the SD-M group or SD-M-PR group, compared with the one in the control group (control vs. SD-M: IRR = 4.22, 95% CI = 1.93 to 9.23**, p-value < 0.001**; control vs. SD-M-PR: IRR = 3.49, 95% CI = 1.92 to 6.37, **p-value < 0.001**, respectively)., The number of newly-tested alters motivated by each index participant was not associated with whether index participants were grouped in SD-M or SD-M-PR (IRR = 1.45, 95% CI = 0.58 to 3.61, **p-value > 0.05**).

**Comment 8:** “At line 191, please make sure it is clear to readers that all participants were required to pay the deposit, not just some of them (or alternatively, please let me know if I have that wrong).”

**Response:** Thanks for the comment, you are right that all participants were required to pay the deposit, not just some of them. We revised the line 195-197 to make it clearer:

“Index participants ordered HIVST kits by paying a refundable deposit for each kit, 100 RMB ($15), which was the equal value of the testing kit and gifts (condoms and lubricants) included in the testing package”.

**Part 2, Response to the comment of Reviewer 1**

**Comment 1:** “Table 2 column headers use the phrase "by each index participant" but it seems to be the total number from ALL index participants. Check and revise throughout as appropriate.”

**Response:** Thanks for the comments. We agree that it is “by all index participants” or “the total number of unique alters motivated by each index participant”. We have revised table 2 accordingly.

**Table 2. Outcome analysis of 309 index participants in China at the end of the study, 2019-2020**

| **Outcomes** |  | **Total number of unique alters motivated by each index participant** | **Total umber of newly-tested alters motivated by each index participant** | **Total number of alters with HIV-reactive results identified by each index participant** |
| --- | --- | --- | --- | --- |
| **Groups, n**  **(Mean, SD)** | Control* (N=102) | 58 (0.57, 0.96) | 16 (0.16, 0.39) | 6 (0.06, 0.24) |
|  | SD-M† (N=103) | 101 (0.98, 1.38) | 42 (0.41, 0.73) | 4 (0.04, 0.19) |
|  | SD-M-PR‡ (N=104) | 185 (1.78, 2.05) | 59 (0.57, 0.91) | 5 (0.05, 0.17) |
| **Mean Difference** | Control (ref) vs SD-M | 0.41 | 0.25 | -0.02 |
|  | Control (ref) vs SD-M-PR | 1.21 | 0.41 | -0.01 |
|  | SD-M (ref) vs  SD-M-PR | 0.80 | 0.16 | 0.01 |
| **Models, IRR/OR (95% CI), p-value** | Control (ref) vs SD-M | 2.98 (1.82, 4.89) *** | 4.22 (1.93, 9.23) *** | 0.79 (0.18, 3.27) |
|  | Control (ref) vs SD-M-PR | 3.26 (2.29, 4.63) *** | 3.49 (1.92, 6.37) *** | 0.79 (0.21, 2.89) |
|  | SD-M (ref) vs SD-M-PR | 1.25 (0.87, 1.81) | 1.45 (0.58, 3.61) | 1.19 (0.25, 6.24) |

Mean refers to each outcome/total number of index participants in each group. *Control refers to the standard secondary distribution group. †SD-M refers to secondary distribution with monetary incentives group. ‡SD-M-PR refers to secondary distribution with monetary incentives plus peer referral group. 0R = odds ratio, IRR = incidence rate ratio. Zero inflated negative binomial regression was used to calculate IRR for outcomes “number of unique alters tested” and “the number of newly-tested alters for HIV”. Logistic regression was used to calculate OR for outcome “Number of alters identified with HIV-reactive results”. IRR or OR were all adjusted for age, income, education, marital status, sexual orientation, and residence. **p < 0.01, ***p < 0.001.

**Comment 2:** “The footnote of Table 2 says "Zero inflated negative binomial regression was used to calculate IRR for outcomes "number of unique alters tested" and "number of newly-tested alters for HIV". Logistic regression was used to calculate OR for outcome "Number of alters identified with HIV-reactive results". Why were different regression methods used for these analyses?”

**Response:** Thanks for the comment, the largest number of the HIV-reactive alters by one index is 1, and thus it obeys bernouli distribution. Therefore, we chose binomial logistic regression model to measure this outcome. The different regression methods were chosen based on the structure of the evaluated data. While the data structure of the outcomes are quite different, we need to use different models to reflect the differences.

**Comment 3:** “The new CEA interpretation has a sentence, "For programs with a budget of more than $7688·30 and a cost-effectiveness threshold of no less than $17.73 per tester..." Where did these numbers come from? The $17.73 does not match numbers given in the table and the total budget seems to be driven by the sample size in the study, not a population. The cost-effectiveness thresholds are somewhat arbitrary; are there any data about other interventions that are more or less costly per person tested that have been adopted to ground these estimates in?”

**Response:** Thanks for the comment and sorry for the confusion. We have deleted this sentence from the discussion point to make the context clearer and easier to follow. We also have updated the Table 4, to make the numbers consistent with the text. We agree that the cost-effectiveness thresholds are somewhat arbitrary and driven by too many factors. In the revised manuscript, we no longer report a threshold.
