# Supplementary material for: Monetary incentives and peer referral in promoting secondary distribution of HIV self-testing among men who have sex with men in China: A randomized controlled trial
Source: PLoS Med. 2022 Feb 14;19(2):e1003928. doi: 10.1371/journal.pmed.1003928 (PMC8887971; doi:10.1371/journal.pmed.1003928)
Supplement: Attachment — Submitted filename: Response letter_19Jan22.docx [file pmed.1003928.s018.docx]

Dear Dr. Davidson,

Thank you for accepting our manuscript for publication on PLOS Medicine. We are glad to share these exciting results with the global audience through PLOS Medicine. We have revised the manuscript according to the comments and further polished it for publication. Please find our point-to-point responses, a marked-up copy of the revised manuscript, and a clean copy of the revised manuscript.

Weiming Tang, Ying Lu and Yuxin Ni on behalf of the team

——————————————————————————————
**Part 1, Response to the comments from the Editors**

**Comment 1:** "Line 22: Please update the phrasing here to more clearly define your primary outcomes in the abstract. For example, "The primary outcomes were the mean number of motivated alters who have photo-verified self-testing per index in each arm, the proportion of first-time HIV testing among alters in each arm, and the proportion of alters with a positive HIV testing result in each arm. These were assessed using zero-inflated negative binomial regression." Please adjust phrasing as required to ensure consistency between the abstract and the main text."

**Response**: Thanks for the comments. We have revised it in the manuscript accordingly.

"The primary outcomes were the total number of motivated alters who have a photo-verified self-testing by index participants in each arm, and the total number of newly-tested alters motivated by index participants in each arm. These were assessed using zero-inflated negative binomial regression to determine the group differences in the total number of alters and the total number of newly tested alters motivated by index participants."

**Comment 2**: "Line 25: Please indicate that these results are mean ± standard deviation."

**Response:** Thanks for the suggestion. We have revised the manuscript.

"The number of unique tested alters motivated by each index was 0.57±0.96 (mean ± standard deviation [SD]) in the control group, compared with 0.98±1.38 in the SD-M group (mean difference [MD]= 0.41), and 1.78±2.05 in the SD-M-PR group (MD=1.21). The number of newly tested alters motivated by each index was 0.16±0.39 (mean ± SD) in the control group, compared with 0.41±0.73 in the SD-M group (MD=0.25), and 0.57±0.91 in the SD-M-PR group (MD=0.41), respectively."

**Comment 3: "**Line 35: Please expand this sentence to state the total number of HIV positive participants and the proportion of first-time HIV testing among alters in each arm (per the outcomes listed in the protocol). Consider including a sentence relating to the third outcome, e.g., "there were similar proportions of alters newly testing positive in the three groups**"**".**”**

**Response:** Thanks for your suggestion. We have added information accordingly in the abstract.

"The proportion of newly-tested testers among alters was 28% in the control group, 42% in the SD-M group, and 32% in the SD-M-PR group. Eighteen testers (3 index participants, 15 alters) tested as HIV-reactive, and the HIV-reactive rates for alters were similar between the three groups."

**Comment 4:** "Please mention the refundable deposit in the abstract."

**Response:** Thanks for the suggestion. We have added deposit information to the abstract.

"Both index participants and alters needed to pay a refundable deposit (USD 15) for ordering a kit."

**Comment 5:** "Line 75: Please include standard deviation in the Author Summary."

**Response:** Thank you for the comment. We have included standard deviation in the Author Summary.

"In the two intervention groups, the number of tested alters motivated by each index was 0.98 (SD=1.38) in the monetary incentives group and 1.78 (SD=2.05) in the monetary incentives plus peer referral group, compared to 0.57 (SD=0.96) in the control group."

**Comment 6:** "Line 84: In the interest of brevity, please remove this bullet point (I feel the following bullet conveys a similar point). "

**Response:** Thank you for the suggestion. We have removed that point in the manuscript.

**Comment 7:** "Line 246: Please update 'Our primary outcomes included to 'Primary outcomes were …'."

**Response:** Thank you for the suggestion. We have revised it.

"Primary outcomes were: 1) the total number of unique tested alters motivated by index participants and 2) the total number of newly-tested alters motivated by index participants for each arm."

**Comment 8**: "Line 256: Please check the numbering of your supplementary file contents – the protocol amendments are located in S4 not S3 (please check throughout)."

**Response:** Thank you for the comment. We have checked the numbering of all supplementary file.

**Comment 9**: "Line 269: Please note CONSORT discourages baseline comparisons. Please ensure these have been consistently removed throughout the manuscript (beyond removing the p-values from Table 1), from e.g. lines 315-16."

**Response:** Thank you for the comment. We have removed the comparison in Table 1 and further clarified it.

"We used Chi-square tests to compare baseline differences between index participants who completed the 3-month follow-up survey and those who did not."

**Comment 10:** "Line 278: 'HIV-reactive results'."

**Response:** Thanks for the comment. We have revised it to "HIV-reactive results".

"We applied logistic regression for secondary outcome analyses, i.e., the total number of alters with HIV-reactive results identified by index participants, by estimating the odds ratio (OR) and its associated 95% CI."

**Comment 11:** “Line 302: Please report <1% rather than 0%.”

**Response:** Thanks for the suggestion. We have revised accordingly in the manuscript.

"One participant (<1%, 1/417) did not sign the informed consents, 89 (21%, 89/417) consented but did not pay the deposit for unknown reasons, and 18 (4%, 18/417) were excluded with reasons due to unexpected technical issues or study inclusion criteria."

**Comment 12:** "Line 322: Please add a space between 'returned' and '209'."

**Response**: Thanks for the suggestion, we have revised accordingly in the manuscript.

"Index participants in the control arm ordered a total of 222 kits, and testers returned 209 test results (returning rate: 94%, 209/222), among which 144 results were from index participants self-testing, and 65 (31%, 65/209) results were from 58 unique alters."

**Comment 13**: "Line 344: As above, this should be Supplementary Table 2 not 1 (please check throughout)."

**Response:** Thanks for the suggestion, we have revised accordingly in the manuscript.

"More information regarding alters' characteristics is listed in Supplementary Table 3."

**Comment 14**: "Line 363: Please remove the comma after the full stop."

**Response****:** Thanks for the comment, we have removed that comma.

**Comment 15:** "Lines 376-377: Please rephrase this sentence for clarity (the wording at line 353-355 is more clear)."

**Response:** Thanks for the suggestion, we have revised accordingly in the manuscript.

"The number of unique alters with HIV-reactive results motivated by each index was not associated with the assignment of index participants to the groups (Table 2)."

**Comment 16**: "Line 475: 'would be a cost-effective strategy'."

**Response**: Thank you for the comment, we have revised the sentence.

"This implied that it would be a cost-effective strategy, given a cost-effectiveness threshold of no less than $13.35 per tester."

**Comment 17**: "478: Please delete the word 'however; from this sentence."

**Response**: Thank you for the comment, the word has been deleted.

**Comment 18: "**Lines 521-523: Please provide a supplementary table with the data to support this statement (participants who were not followed up had similar social demographic characteristics as those who had completed the three-month follow-up survey) and add a reference to it in your Results section. Please re-number all supplementary tables as required.**"**

**Response:** Thanks for pointing this out. We have added the related information in the results, provided a supplementary table, and re-numbered supplementary tables accordingly.

"There was no significant difference (p-value > 0.05) in the social demographic characteristics between index participants who completed the 3-month follow-up survey and those who did not (Supplementary Table 1)."

**S1 Table. Characteristics of the index participants regarding 3-month follow-up survey completion, 2019-2020 (N=309)**

|  | **Index participants completed the 3-month follow-up survey** | |
| --- | --- | --- |
|  | **Yes**  **(n=293)** | **No**  **(n=16)** |
| **Age (years)** |  |  |
| *≤ 30* | 189 (65%) | 13 (81%) |
| *＞30* | 104 (35%) | 3 (19%) |
| **Education** |  |  |
| *High school or below* | 56 (19%) | 6 (38%) |
| *College* | 211 (72%) | 10 (62%) |
| *Master’s degree or above* | 26 (9%) | 0 (0%) |
| **Monthly Income (USD, $)** |  |  |
| *<225* | 23 (8%) | 1 (6%) |
| *225-450* | 16 (5%) | 3 (19%) |
| *451-750* | 69 (23%) | 5 (31%) |
| *751-1200* | 100 (34%) | 3 (19%) |
| *>1200* | 85 (29%) | 4 (25%) |
| **Sexual Orientation** |  |  |
| *Gay* | 212 (72%) | 9 (56%) |
| *Others*\| | 81 (28%) | 7 (44%) |
| **Marital Status** |  |  |
| *Single* | 246 (84%) | 15 (94%) |
| *Engaged or married* | 37 (13%) | 0 (0%) |
| *Separated or divorced* | 10 (3%) | 1 (6%) |
| **Residence** |  |  |
| *Guangdong province* | 201 (68%) | 10 (62%) |
| *Others* | 92 (32%) | 6 (38%) |

Data were presented as n (%). |Others include heterosexual, bisexual, and not sure.

**Comment 19:** "Table 2: Unless I am missing it, I cannot see any data to which the footnote labelled ** applies (p<0.001).**"**

**Response:** Sorry for the confusion and thanks for pointing this out, we have revised the footnote accordingly.

"Logistic regression was used to calculate OR for outcome "Number of alters identified with HIV-reactive results". IRR or OR were all adjusted for age, income, education, marital status, sexual orientation, and residence. ***p < 0.001.”

**Comment 20:** "Supplementary Table 3: Please include the full excel sheet of cost items in your supplementary material as a study author cannot be the point of contact for data requests."

**Response:** Thanks for the suggestion, we will submit the cost data excel sheet accordingly.

**Comment 21**: "We have noticed that you have cited Supporting Information file ‘S5 file’ in the manuscript file. However, there are no corresponding files uploaded to the submission. Please ensure that all files are present in the file inventory."

**Response:** Thanks for pointing this out, and sorry for the confusion. We have removed the “S5 file” from the manuscript uploaded the compressed de-identified study data separately for submission.

**Part 2, Response to the comment of Reviewer 1**

**Comment 1: "**1) Table 2: while the authors stated in their response letter that the headers should refer to "by all participants" rather than "by each participant", but the header was revised to read "Total number of unique alters motivated by each index participant". Recommend revising to have each --> all or "total number of unique alters motivated by participants". As is, it is unclear. (Also recommend a spell check to catch typos like "umber" in lieu of "number" in header).**"**

**Response:** Thanks for the suggestions. We revised all primary outcomes as "the total number of unique alters motivated by index participants" and "the total number of newly-tested alters motivated by index participants".

**Table 2. Outcome analysis of 309 index participants in China at the end of the study, 2019-2020**

| **Outcomes** |  | **Total number of unique alters motivated by index participants** | **Total number of newly-tested alters motivated by index participants** | **Total number of alters with HIV-reactive results identified by index participants** |
| --- | --- | --- | --- | --- |
| **Groups, n**  **(Mean, SD)** | Control* (N=102) | 58 (0.57, 0.96) | 16 (0.16, 0.39) | 6 (0.06, 0.24) |
|  | SD-M† (N=103) | 101 (0.98, 1.38) | 42 (0.41, 0.73) | 4 (0.04, 0.19) |
|  | SD-M-PR‡ (N=104) | 185 (1.78, 2.05) | 59 (0.57, 0.91) | 5 (0.05, 0.17) |
| **Mean Difference** | Control (ref) vs SD-M | 0.41 | 0.25 | -0.02 |
|  | Control (ref) vs SD-M-PR | 1.21 | 0.41 | -0.01 |
|  | SD-M (ref) vs  SD-M-PR | 0.80 | 0.16 | 0.01 |
| **Models, IRR/OR (95% CI), p-value** | Control (ref) vs SD-M | 2.98 (1.82, 4.89) *** | 4.22 (1.93, 9.23) *** | 0.79 (0.18, 3.27) |
|  | Control (ref) vs SD-M-PR | 3.26 (2.29, 4.63) *** | 3.49 (1.92, 6.37) *** | 0.79 (0.21, 2.89) |
|  | SD-M (ref) vs SD-M-PR | 1.25 (0.87, 1.81) | 1.45 (0.58, 3.61) | 1.19 (0.25, 6.24) |

Mean refers to each outcome/total number of index participants in each group. *Control refers to the standard secondary distribution group. †SD-M refers to secondary distribution with monetary incentives group. ‡SD-M-PR refers to secondary distribution with monetary incentives plus peer referral group. 0R = odds ratio, IRR = incidence rate ratio. Zero inflated negative binomial regression was used to calculate IRR for outcomes "the total number of unique alters motivated by index participants" and "the total number of newly-tested alters motivated by index participants". Logistic regression was used to calculate OR for outcome "the total number of alters with HIV-reactive results identified by index participants". IRR or OR were all adjusted for age, income, education, marital status, sexual orientation, and residence. ***p < 0.001.

**Comment 2:** "2) Table 4 has been flipped in orientation for some reason, and the resultant table is not a traditional CEA format and is less clear. If the authors prefer to retain as is for a particular reason, that's fine, but it is an unnecessary change that does not aid in clarity."

**Response:** Thanks for the comment. We flipped Table 4 to show the comparable results of the SD-M-PR arm to the control arm in support of the text interpretation, as the original table, which was in traditional CEA format, was deemed correct but lacked the aforementioned comparison Reviewer 2 in their previous comments. We agree that the current format does not aid in clarity, but since the added information is not omittable and the clarity is not substantially reduced, we have decided to retain this flipped table format.

**Comment 3:** "3) The authors state in their response letter that they've removed the thresholds for cost-effectiveness, but this partially remains in the manuscript, along with cost-effective language. Recommend a careful re-read of every section of the manuscript that deals with the health economics evaluation in full with attention to prior review comments to make changes consistent and accurate throughout instead of partially changed and with some retained errors in reach revision. I did not re-read every section in careful detail to catch each of these errors beyond the first one noted"

**Response:** Thanks for the comment and suggestion. We have removed the manuscript's remaining content about cost-effectiveness thresholds (page 23). And we have also re-read the health economics evaluation sections throughout the manuscript carefully to make sure the cost-effectiveness-related content in the table and the text were consistent and accurate after all the changes we made.
