# Supplementary material for: Monetary incentives and peer referral in promoting secondary distribution of HIV self-testing among men who have sex with men in China: A randomized controlled trial
Source: PLoS Med. 2022 Feb 14;19(2):e1003928. doi: 10.1371/journal.pmed.1003928 (PMC8887971; doi:10.1371/journal.pmed.1003928)
Supplement: Attachment — Submitted filename: Reviewer Comments July 2021-submit.pdf [file pmed.1003928.s014.pdf]

REVIEW

Summary

This publication addresses an important field: improving HIV testing in a high risk group – MSM in a Chinese context. It utilizes approaches that have been shown to be successful in other areas of HIV prevention and care to be directly applied to the MSM context. In addition there is provision of graduation of the intervention by employing a three-arm design to explore synergy that may be achieved from two distinct strategies. It is highly relevant in the context of COVID-19 that approaches that can be employed remotely be systematically assessed. The findings of the paper have practical relevance to policy making and practice. One important omission is the lack of clear description of the background stigma that may exists in the population studied that has been seen in other areas where HIV partner tracing has been studied and how it relates to the adverse effects of the intervention.

Rationale for the study: The study is based on the fact that only about half of MSM in China are aware of their HIV status despite a known high prevalence (>5%) in previous studies. The second justification is that COVID has limited face to face services that would routinely be used to improve the yield of testing services leaving few options available. While HIV ST approaches have been successfully in increasing the reach of testing and linkage to care including ART initiation most studies are based in Africa. The author should indicate that there is a reasonable expectation that this will be the case in the context of China which has very different sociocultural context and levels of HIV associated stigma.

Methods:

Outcomes definition: The primary outcome is provided in two ways:

a). the mean number of unique alters motivated (equivalent to contacts successfully tested through the efforts of the index) computed by study arm.

b). the number of newly tested alters motivated by each index case.

Secondary outcomes are provided as the number of newly tested alters who returned a positive HIV or syphilis test. Other aspects of the secondary outcome are less clear. Economic analysis are perform to determine the costs including the cost per person tested and the cost of each trial arm.

Sample size: This is provided based on findings from a pilot study. The standard assumptions of power (> 80%), alpha (0.05) for the minimum detectable effect size are clearly provided.

Randomization: This was done by computer generated randomization. The author should comment on whether block sizes with variable block sizes or otherwise was employed.

Blinding: Study participants and staff were unblinded but the statisticians analysing the data were blinded.

Allocation concealment: This is not described and should be included.

Results: The primary outcome is clearly reported as the mean number of tested alters motivated by index participants **per arm** (0.57, 0.98 and 1.98) in the control and two intervention arms respectively. The intervention arms both had higher rates of HIV testing than the control.

The **mean difference** and confidence intervals between the control and each of the two intervention arms are provided as required. The second presentation of the primary outcome (mean number of newly tested motivated alters **per index case** is also clearly provided along with the mean differences. Standard deviations and risk estimates are provided. The superiority of the third intervention arm (SD-M-PR) over the second arm (SD-M) is also provided as part of the primary outcomes.

Secondary outcomes: The HIV positivity rate is reported and did not differ between arms. The economic costs are described and compared between arms and show that the interventions had a lower cost per alter tested which points to cost-efficiency.

Harms: There is no deliberate reporting of harms. Specifically the authors should indicate whether or not there were any cases of social harms related to HIV testing and diagnosis.

Discussion: This reflects the importance of the study findings. An additional limitation is generalizability beyond the period of COVID with its mitigation measures. For instance it is unclear to what extend the same monetary incentive will hold after economic recovery.

Trial registration: This is provided as ChiCTR 1900025433)

1) Outcomes (Item 6a)
Completely defined pre-specified primary outcome measure including how and when it was assessed
Is it clear (1) what the primary outcome is (usually the one used in the sample size calculation), (2) how it was measured (if relevant; e.g. which score used), (3) at what time point, and (4) what the analysis metric was (e.g. change from baseline, final value)?

2) Sample size (Item 7a)
How sample size was determined
Is there a clear description of how the sample size was determined, including (1) the estimated outcomes in each group; (2) the α (type I) error level; (3) the statistical power (or the β (type II) error level); and (4) for continuous outcomes, the standard deviation of the measurements?

3) Sequence generation (Item 8a)
Method used to generate random allocation sequence
Does the description make it clear if the "assigned intervention is determined by a chance process and cannot be predicted"?

4) Allocation concealment (Item 9)
Mechanism used to implement random allocation sequence (such as sequentially numbered containers), describing any steps taken to conceal the sequence until interventions were assigned
Is it clear how the care provider enrolling participants was made ignorant of the next assignment in the sequence (different from blinding)? Possible methods can rely on centralised or "third-party" assignment (i.e., use of a central telephone randomisation system, automated assignment system, sealed containers).

5) Blinding (Item 11a)
If done, who was blinded after assignment to interventions (for example, participants, care providers, those assessing outcomes)
Is it clear if (1) healthcare providers, (2) patients, and (3) outcome assessors are blinded to the intervention? General terms such as "double-blind" without further specifications should be avoided.

6) Outcomes and estimation (Item 17a/b)
For the primary outcome, results for each group, and the estimated effect size and its precision (such as 95% confidence intervals)
Is the estimated effect size and its precision (such as standard deviation or 95% confidence intervals) for each treatment arm reported? When the primary outcome is binary, both the relative effect (risk ratio, relative risk) or odds ratio) and the absolute effect (risk difference) should be reported with confidence intervals.

7) Harms (Items 19)
All important harms or unintended effects in each group
Is the number of affected persons in each group, the severity grade (if relevant) and the absolute risk (e.g. frequency of incidence) reported? Are the number of serious, life threatening events and deaths reported? If no adverse event occurred this should be clearly

stated.

8) Registration (Item 23)

Registration number and name of trial registry

Is the registry and the registration number reported? If the trial was not registered, it should be explained why.

9) Protocol (Item 24)

Where trial protocol can be accessed

Is it stated where the trial protocol can be assessed (e.g. published, supplementary file, repository, directly from author, confidential and therefore not available)?

10) Funding (Item 25)

Sources of funding and other support (such as supply of drugs) and role of funders

Are (1) the funding sources, and (2) the role of the funder(s) described?
